# Introducing difference recurrence relations for faster semi-global alignment of long sequences

**DOI:** 10.1186/s12859-018-2014-8

**Published:** 2018-02-19

**Authors:** Hajime Suzuki, Masahiro Kasahara

**Affiliations:** Department of Computational Biology and Medical Sciences, Graduate School of Frontier Sciences, the University of Tokyo, Kashiwa City, Chiba Japan

**Keywords:** Sequence analysis, Alignment, Long read

## Abstract

**Background:**

The read length of single-molecule DNA sequencers is reaching 1 Mb. Popular alignment software tools widely used for analyzing such long reads often take advantage of single-instruction multiple-data (SIMD) operations to accelerate calculation of dynamic programming (DP) matrices in the Smith–Waterman–Gotoh (SWG) algorithm with a fixed alignment start position at the origin. Nonetheless, 16-bit or 32-bit integers are necessary for storing the values in a DP matrix when sequences to be aligned are long; this situation hampers the use of the full SIMD width of modern processors.

**Results:**

We proposed a faster semi-global alignment algorithm, “difference recurrence relations,” that runs more rapidly than the state-of-the-art algorithm by a factor of 2.1. Instead of calculating and storing all the values in a DP matrix directly, our algorithm computes and stores mainly the differences between the values of adjacent cells in the matrix. Although the SWG algorithm and our algorithm can output exactly the same result, our algorithm mainly involves 8-bit integer operations, enabling us to exploit the full width of SIMD operations (e.g., 32) on modern processors. We also developed a library, libgaba, so that developers can easily integrate our algorithm into alignment programs.

**Conclusions:**

Our novel algorithm and optimized library implementation will facilitate accelerating nucleotide long-read analysis algorithms that use pairwise alignment stages. The library is implemented in the C programming language and available at https://github.com/ocxtal/libgaba.

**Electronic supplementary material:**

The online version of this article (10.1186/s12859-018-2014-8) contains supplementary material, which is available to authorized users.

## Background

Recent advances in single-molecule sequencers enabled researchers to obtain much longer reads than those offered by Sanger sequencers. Since Pacific Biosciences released its first real-time single-molecule sequencer, PacBio RS, in 2010, the read length of single-molecule sequencers has been increasing. The latest Sequel sequencer can yield reads longer than 20 kbp. MinION sequencers with the R9.4 chemistry released by Oxford Nanopore Technology are reported to generate a read nearly a megabase long [1]. The output of these sequencers is typically aligned against a reference genome for downstream analyses such as quantification of gene expression levels and identification of isoforms [2] and structural variants [3, 4]. Another major application of long reads is *de novo* assembly, where whole-genome shotgun reads are aligned with each other, and then contigs and their consensus sequences are built [5, 6]. Because sequence alignment is one of the most fundamental methods in all kinds of genomic analyses, it is important to develop a fast and efficient sequence alignment algorithm. Local alignments of nucleotide sequences are often identified by popular general-purpose alignment tools such as BLAST [7], BWA-MEM [8], or LAST [9], but there are faster alignment algorithms that fully support long reads from single-molecule sequencers. For example, BLASR [10], DALIGNER [11], and GraphMap [12] have shown a better balance among sensitivity, alignment quality, and computation time for long reads with abundant indels (insertions and deletions). Considering that the throughput of long-read sequencers is expected to double annually in the next few years as the vendors claim, it is conceivable that investigators will obtain terabases per day from a single sequencing instrument. Therefore, the sensitivity and speed of the current alignment algorithms still need to be improved, especially for *de novo* assembly, which requires huge computation time for all-versus-all comparison of reads.

Pairwise alignment of nucleotide sequences is often calculated by the Smith–Waterman–Gotoh (SWG) algorithm [13, 14] or its variants. The original SWG algorithm is usually used in combination with a heuristic called the “seed-and-extend” strategy in practical applications. The seed-and-extend strategy first detects a seed(s), which is an exact-matching sequence or a near-exact-matching pattern shared between two sequences, and then calculates a detailed pairwise alignment around the seed. To find a pairwise alignment around the seed, the “semi-global alignment” algorithm, in which one end of the alignment is fixed and the other end is open, is often applied. One of the efficient methods for calculating the semi-global alignment is the X-drop cutoff algorithm in BLAST, which terminates a search when scores drop by a certain amount. Similar algorithms are implemented in BLASTZ [15] and LAST. Another well-known method for reducing computation time is called “banded DP” [16], by which researchers calculate only a part of DP cells within a threshold distance from the diagonal line in the DP matrix.

Another line of research for accelerating pairwise alignment involves data level parallel instructions, also known as single-instruction multiple-data (SIMD) instructions. These methods can carry out a vector operation in a single instruction and thus can accelerate the SWG algorithm. Such examples include methods developed by Wozniak [17], Rognes et al. [18], and Farrar [19]. Farrar’s striped DP algorithm has been the fastest for protein sequences and short nucleotide sequences, and that is the reason why Farrar’s algorithm is adopted in the most popular alignment programs such as BWA [20], Bowtie2 [21], and HMMER [22] and alignment libraries such as SSW [23] and Parasail [24].

Nonetheless, how to combine the idea of using SIMD and the notion of reducing the search space in a semi-global alignment for further acceleration of the semi-global alignment is not obvious, especially when two sequences to be aligned contain abundant indel errors introduced by single-molecule sequencers. We need prohibitively large band width to ensure that the optimal alignment path is contained in the band of banded DP, given that the read length is approaching 1 megabase. To this end, we proposed a SIMD-enabled adaptive banded DP algorithm [25] with constant band width for aligning reads with abundant but stochastic indels. The algorithm adaptively defines the band such that the band width is a constant, and still the optimal alignment path is contained in the band at a high probability at typical settings for single-molecule sequencing reads.

A new problem that we uncovered in the adaptive banded DP algorithm is that the number of parallelisms (i.e., vector width) in SIMD operations is often limited to a half or a quarter of the number of parallelisms intrinsic to hardware. For example, recent Intel CPUs with Advanced Vector eXtension 2 (AVX2) have SIMD operations of 32 integers of 8 bits; therefore, we expect that 32 cells in a DP matrix should be computed in a single operation. As the read length increases, however, the absolute values of cells in the DP matrix increase; the number of bits required for storing the value of a single cell becomes 16 or even 32, which means that we cannot use 8-bit integers anymore for a DP matrix. If we use 32-bit integers, then vector width is limited to 8 because a single AVX2 register can hold only 8 values of 32-bit integers. Note that the problem persists even if we give up the adaptive banded DP algorithm.

To use 8-bit integers mostly for a DP matrix, we propose a “difference recurrence relation” that is a variant of semi-global alignment DP with an affine gap penalty, but most computations involve only 8-bit integers under reasonable conditions. Our contribution is threefold: (1) we propose new recurrences for the semi-global alignment suitable for 8-bit SIMD operations, (2) we present several implementation techniques for further acceleration of computation of the semi-global alignment, and (3) we developed a library that is easy to integrate with genome analysis tools. Our algorithm can be considered a generalization of score parameters in the existing bit-parallel algorithms such as Myers’ edit distance algorithm [26] and Hyyrö’s longest common substring algorithm [27], which inspired our idea. We demonstrate the efficiency of our algorithm on real long reads. We compared our new algorithm with the fastest algorithm for the semi-global alignment of long reads (i.e., adaptive banded DP with SIMD instructions) and several baseline algorithms that use either SIMD instructions or bit parallelisms, showing that our new algorithm runs 2.1-fold faster than does its counterpart with wider integers.

## Methods

### The semi-global DP algorithm

Equation 1 shown below is the definition of the semi-global DP algorithm we use throughout the paper. It is a trivial variant of the original SWG algorithm [13, 14]. Although we focus on the semi-global alignment algorithm, the same argument holds for the global alignment algorithm.

1 $$\begin{array}{*{20}l} {}S[\!i, j] &\,=\, \left\{\!\! \begin{array}{ll} 0 & (i \,=\, 0, j \,=\, 0) \\ -G_{o_{V}} - j \cdot G_{e_{V}} & (i \,=\, 0, j \!\neq\! 0) \\ -G_{o_{H}} - i \cdot G_{e_{H}} & (i \!\neq\! 0, j \,=\, 0) \\ \max \left\{\! \begin{array}{l} S[\!i - 1, j - 1] + s(a_{i - 1}, b_{j - 1}) \\ E[\!i - 1, j] - G_{e_{H}} \\ F[\!i, j - 1] - G_{e_{V}} \end{array} \right. & (i \!\neq\! 0, j \!\neq\! 0) \end{array} \right.  \\ {}E[\!i, j] &\,=\, \left\{\!\! \begin{array}{ll} -G_{o_{H}} - i \cdot G_{e_{H}} & (j = 0) \\ -\inf & (i = 0) \\ \max \left\{ \begin{array}{l} S[\!i, j] - G_{o_{H}} \\ E[\!i - 1, j] - G_{e_{H}} \end{array} \right. & (i \neq 0, j \neq 0) \end{array} \right.  \\ {}F[\!i, j] &\,=\, \left\{\!\! \begin{array}{ll} -G_{o_{V}} - j \cdot G_{e_{V}} & (i = 0) \\ -\inf & (j = 0) \\ \max \left\{ \begin{array}{l} S[\!i, j] - G_{o_{V}} \\ F[\!i, j - 1] - G_{e_{V}} \end{array} \right. & (i \neq 0, j \neq 0) \end{array} \right.  \\  \end{array} $$

Two input sequences are represented as *a*=*a*_*o*_*a*_1_...*a*_|*a*|−1_ and *b*=*b*_0_*b*_1_...*b*_|*b*|−1_ over alphabet *Σ*={*A*,*C*,*G*,*T*}. The substitution matrix is described as function *s*(*x*,*y*), where *x*,*y*∈ *Σ*. *M* is the maximum value in the substitution matrix, and hence *M*= max*p*,*q*∈*Σs*(*p*,*q*). We assume that *M* is non-negative and that $\min _{p, q \in \Sigma } s(p, q) > - (G_{o_{H}} + G_{e_{H}} + G_{o_{V}} + G_{e_{V}})$. The gap penalty function is expressed in linear form: *G*(*k*)=*G*_*o*_+*k*·*G*_*e*_, where *k* is the length of a contiguous gap region and *G*_*o*_ (≥ 0) and *G*_*e*_ (> 0) are gap-open and gap-extension penalties. In the formulae, the vertical and horizontal gap penalties are distinguished by *H* and *V* subscripts, respectively, although they often have the same value in real-world applications. The cell at the origin (0,0) is initialized with 0, and the other edge cells at *i*=0 and at *j*=0 are calculated as the penalty score for a contiguous gap from the origin. The traceback starts from the cell with the maximum value in the DP matrix and terminates at the origin; this approach ensures that the left (5’-) end of the alignment is fixed at the origin, and that the right (3’-) end is open.

### Naïve difference recurrence relations

**Fig. 1:**
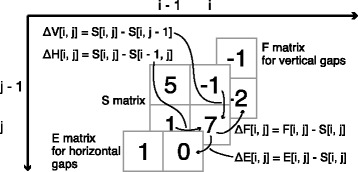
Four difference matrices. *ΔH* and *ΔV* represent the differences between horizontally and vertically adjacent cells in *S*. *ΔE* and *ΔF* are the differences between a pair of cells at the same *i*,*j*-coordinates in *E* and *S*, and *F* and *S*, respectively

Next, we explain a naïve version of our algorithm to navigate readers smoothly to the final version of the algorithm that we propose. In the original SWG algorithm, the recurrence contains a comparison operation with an absolute value; in every cell in the DP matrix, the score is compared with zero, which is an absolute value. In contrast, the semi-global alignment algorithm and its variants do not have any comparison operation with an absolute value, so that the recurrences can be transformed into the ones in difference form, where the matrix can be perfectly reconstructed from a set of difference values between vertically or horizontally adjacent cells and the initial value (i.e., 0) of the cell at the origin, (0,0). We introduce four difference matrices, *ΔH* for *i*≥1, *ΔV* for *j*≥1, and *ΔE* and *ΔF* across the whole matrix as follows. Figure 1 illustrates these difference matrices.

$$\begin{array}{*{20}l} \Delta H[\!i, j] &= S[\!i, j] - S[\!i - 1, j] & (i \geq 1)  \\ \Delta V[\!i, j] &= S[\!i, j] - S[\!i, j - 1] & (j \geq 1)  \\ \Delta E[\!i, j] &= E[\!i, j] - S[\!i, j]  \\ \Delta F[\!i, j] &= F[\!i, j] - S[\!i, j]  \end{array} $$

With these four difference matrices, the recurrences for the original semi-global alignment can be transformed into those in difference form. To simplify the recurrences, we introduce intermediate variable *A*[ *i*,*j*], which represents diagonal difference *S*[ *i*,*j*]−*S*[ *i*−1,*j*−1]. Then, the recurrences can be expressed as in Eq. 2 (where *i*≥1 and *j*≥1). Note that the right-hand sides of the recurrences keep symmetry with respect to the two coordinates, *i* and *j*, as those in the original SWG algorithm do. The complete process of derivation of the difference recurrences is described in Additional file 1: Section S1.1.

2 $$\begin{array}{*{20}l} A[\!i, j] &= \max \left\{ \begin{array}{l} s(a_{i - 1}, b_{j - 1}) \\ \Delta E[\!i - 1, j] + \Delta V[\!i - 1, j] - G_{e_{H}} \\ \Delta F[\!i, j - 1] + \Delta H[\!i, j - 1] - G_{e_{V}} \end{array} \right.  \\ \Delta H[\!i, j] &= A[\!i, j] - \Delta V[\!i - 1, j]  \\ \Delta V[\!i, j] &= A[\!i, j] - \Delta H[\!i, j - 1]  \\ \Delta E[\!i, j] &= \max \left\{ \begin{array}{l} - G_{o_{H}} \\ \Delta E[\!i - 1, j] - \Delta H[\!i, j] - G_{e_{H}} \end{array} \right.  \\ \Delta F[\!i, j] &= \max \left\{ \begin{array}{l} - G_{o_{V}} \\ \Delta F[\!i, j - 1] - \Delta V[\!i, j] - G_{e_{V}} \end{array} \right.  \\  \end{array} $$

The initial conditions for the recurrences (i.e., the cell values in the column *i*=0 and the row *j*=0) will be shown shortly. The differences from − inf are clipped to $-G_{o_{H}}$ and $-G_{o_{V}}$ for *ΔE* and *ΔF*, respectively, without a loss of generality. This clipping makes it easier to compute the values of cells using a small integer type such as 8-bit integer, while this clipping does not cause a gap-penalization error at the edges as pointed out by Rognes [28] because it guarantees that $E[0, j] - G_{e_{H}} \leq S\,[\!0, j] - G_{o_{H}} - G_{e_{H}}$ and $F\,[\!i, 0] - G_{e_{V}} \leq S\,[\!i, 0] - G_{o_{V}} - G_{e_{V}}$ for the first update of *ΔE* (where *i*=1) and *ΔF* (*j*=1).

$$\begin{array}{*{20}l} \Delta H[\!i, j] &= \left\{ \ \begin{array}{l} G_{o_{H}} + G_{e_{H}} \\ G_{e_{H}} \end{array} \right. &\begin{array}{l} (i = 1, j = 0) \\ (i \geq 2, j = 0) \end{array}  \\ \Delta V[\!i, j] &= \left\{ \ \begin{array}{l} G_{o_{V}} + G_{e_{V}} \\ G_{e_{V}} \end{array} \right. &\begin{array}{l} (i = 0, j = 1) \\ (i = 0, j \geq 2) \end{array}  \\ \Delta E[\!i, j] &= \left\{ \begin{array}{l} 0 \\ -G_{o_{H}} \end{array} \right. &\begin{array}{l} (j = 0) \\ (i = 0) \end{array}  \\ \Delta F[\!i, j] &= \left\{ \begin{array}{l} 0 \\ -G_{o_{V}} \end{array} \right. &\begin{array}{l} (i = 0) \\ (j = 0) \end{array}  \end{array} $$

The difference values are bounded by certain ranges that can be computed from the gap penalty scores and *M* (the maximum value in the substitution matrix) when the initial conditions are defined as above. This property enables us to use integers of a smaller number of bits for calculating and storing the difference values. For example, a 4-bit signed integer is sufficient when *M*=2, $G_{o_{H}} = G_{o_{V}} = 4$, and $G_{e_{H}} = G_{e_{V}} = 1$. The proof of the bounding formulae shown below is provided in Additional file 1: Section S1.2.

$$\begin{array}{*{20}l} -G_{o_{H}} - G_{e_{H}} &\leq \Delta H \leq M + G_{o_{V}} + G_{e_{V}}  \\ -G_{o_{V}} - G_{e_{V}} &\leq \Delta V \leq M + G_{o_{H}} + G_{e_{H}}  \\ -G_{o_{H}} &\leq \Delta E \leq 0  \\ -G_{o_{V}} &\leq \Delta F \leq 0  \end{array} $$

The difference recurrence relations, under the proper initial conditions, do not lose any information from the original recurrences (hereafter: nondifference recurrences); therefore, we can obtain alignments exactly the same as those produced by the nondifference recurrences. The use of smaller integer types allows us not only to compute values in the DP matrix faster but also to reduce the memory requirements because we need smaller memory for storing the DP matrix. Arbitrarily long alignments can be computed using only integers of a small number of bits, aside from a small part of the DP matrix needs to be stored in absolute values to find the cell with the maximum value (score). This method enables us to take advantage of the full width of SIMD operations on modern processors, which is 16 for Streaming SIMD Extension 2 (SSE2) and 32 for AVX2. In contrast, Farrar [19] suggests retrying to fill the DP matrix with a larger integer type when a value in the DP matrix overflows.

### The proposed algorithm

The difference recurrence relations that we proposed in the previous subsection are already suitable for reducing computation time, but we tried to further optimize the recurrences. First, we transformed *ΔE*, *ΔV*, *ΔF*, and *ΔH* into four new matrices, *ΔH*_*G*_, *ΔV*_*G*_, *ΔEG*′, and *ΔFG*′, which are defined as follows. Note that *ΔE*, *ΔV*, *ΔF*, and *ΔH* can be calculated from the new matrices; therefore, calculating the new matrices is mathematically equivalent to calculating *ΔE*, *ΔV*, *ΔF*, and *ΔH*. We also defined *s*_*G*_ and *A*_*G*_ as a substitution matrix and an intermediate matrix with offsets, respectively. *s*_*G*_ can be precomputed so that introducing *s*_*G*_ does not increase computation time.

$$\begin{array}{*{20}l} A_{G}[\!i, j] &= A[\!i, j] + G_{o_{H}} + G_{e_{H}} + G_{o_{V}} + G_{e_{V}}  \\ \Delta H_{G}[\!i, j] &= \Delta H[\!i, j] + G_{o_{H}} + G_{e_{H}}  \\ \Delta V_{G}[\!i, j] &= \Delta V[\!i, j] + G_{o_{V}} + G_{e_{V}}  \\ \Delta E'_{G}[\!i, j] &= \Delta E[\!i, j] + \Delta V[\!i, j] + G_{o_{H}} + G_{o_{V}} + G_{e_{V}}  \\ \Delta F'_{G}[\!i, j] &= \Delta F[\!i, j] + \Delta H[\!i, j] + G_{o_{V}} + G_{o_{H}} + G_{e_{H}}  \\ s_{G}(x, y) &= s(x, y) + G_{o_{H}} + G_{e_{H}} + G_{o_{V}} + G_{e_{V}}  \end{array} $$

With these definitions, the recurrence relations can be written as in Eq. 3.

3 $$\begin{array}{*{20}l} {}A_{G}[\!i, j] &= \max \left\{ \begin{array}{l} s_{G}(a_{i - 1}, b_{j - 1})\\ \Delta E'_{G}[\!i - 1, j] \\ \Delta F'_{G}[\!i, j - 1] \end{array} \right.  \\ {}\Delta H_{G}[\!i, j] &= A_{G}[\!i, j] - \Delta V_{G}[\!i - 1, j]  \\ {}\Delta V_{G}[\!i, j] &= A_{G}[\!i, j] - \Delta H_{G}[\!i, j - 1]  \\ {}\Delta E'_{G}[\!i, j] &= \max \left\{ \begin{array}{l} A_{G}[\!i, j] \\ \Delta E'_{G}[\!i - 1, j] + G_{o_{H}} \end{array} \right\} - \Delta H_{G}[\!i, j - 1]  \\ {}\Delta F'_{G}[\!i, j] &= \max \left\{ \begin{array}{l} A_{G}[\!i, j] \\ \Delta F'_{G}[\!i, j - 1] + G_{o_{V}} \end{array} \right\} - \Delta V_{G}[\!i - 1, j]  \\  \end{array} $$

The initial conditions for the new matrices are not shown because they are trivial; they are simply the sum of the gap penalties and the initial values in the original matrices. The bounding formulae for the values in the new difference matrices are shown below (see Additional file 1: Sections S1.3 and S1.4 for more details). In the new bounding formulae, the lower bounds are all zero and the upper bounds are a single constant; the values in the new matrices can be stored as an array of unsigned integers.

$$\begin{array}{*{20}l}{} 0 &\leq \Delta H_{G}, \Delta V_{G}, \Delta E'_{G}, \Delta F'_{G} \leq M \,+\, G_{o_{H}} \,+\, G_{e_{H}} \,+\, G_{o_{V}} + G_{e_{V}}  \end{array} $$

Lastly, we investigated the critical path in the recurrences. The length of a critical path is defined as the length of the longest operation dependency chain, where unit operations are basic binary operations such as addition, subtraction, and maximum. The critical path length of the new difference recurrences is reduced to 4 from 8 in the original difference recurrences and even from 5 in the original nondifference semi-global alignment algorithm. The shorter critical path is preferred on modern processors that can perform multiple arithmetic operations in a single clock cycle because independent operations can be executed in parallel by the superscalar instruction execution mechanism.

### Relation to other DP algorithms

The difference recurrence relations are interpreted as a generalization of the existing approximate string-matching algorithms as mentioned in the background. Certain edit distance algorithms and longest common substring (LCS) algorithms express the DP matrix in difference form. The negated *ΔH* and *ΔV* in this paper are equivalent to *Δh* and *Δv* in the article by Myers [26] if the substitution matrix is defined as *s*(*x*,*y*)=0 when *x*=*y* and −1 otherwise, and gap penalties $G_{o_{H}} = G_{o_{V}} = 0$ and $G_{e_{H}} = G_{e_{V}} = 1$. Similarly, *ΔV*[ *i*,*j*] in this paper is equivalent to *V*_*j*_[ *i*] in the bit-parallel LCS algorithm authored by Hyyrö [27] when the substitution matrix is defined as *s*(*x*,*y*)=1 when *x*=*y* and 0 otherwise, and all the gap penalties are zero. Further explanation is provided in the LCS paper describing the conversion of bit variables between the three existing algorithms designed by Allison and Dix [29], by Crochemore et al. [30], and Hyyrö [27]. We also should say that the bit-parallel global alignment algorithm proposed by Loving et al. [31] was the first algorithm that adopted difference recurrences for the SWG algorithm with a linear gap penalty. Our algorithm can be considered a generalization of theirs with an affine gap penalty and maintenance of the symmetry in relation to the coordinates, *i* and *j*.

### Library implementation

We implemented our algorithm as an independent library, libgaba, so that developers can easily integrate our algorithm into alignment tools or other genome analysis software. It is implemented purely in the C language so that it can be called from virtually any programming language. Although our difference recurrences can be applied to global alignments or any variants of semi-global alignments in theory, libgaba is designed specifically for computing semi-global alignment by means of the adaptive banded DP algorithm, because we believe that the combination of our algorithm with the banded DP algorithm takes the best balance between speed and sensitivity for long-read alignment.

**Fig. 2:**
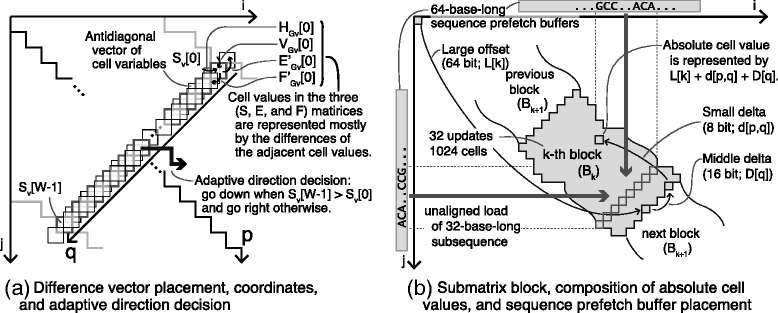
**a** A schematic view of vectors, coordinates, and an adaptive band in the proposed algorithm: Vectors have parallelisms in the antidiagonal direction. *W* denotes the band width in adaptive banded DP. A set of four vectors, $\Delta H_{G_{V}}$, $\Delta V_{G_{V}}$, $\Delta E'_{G_{V}}$, and $\Delta F'_{G_{V}}$, retains the forefront vectors in the four difference DP matrices *ΔH*_*G*_, *ΔV*_*G*_, *ΔEG*′, and *ΔFG*′. Two additional coordinates, *p* and *q*, are introduced to index vectors and vector lanes in addition to the horizontal and vertical coordinates, *i* and *j*, in the DP matrix. The *p* coordinate, defined as *p*=*i*+*j*, specifies the location of the vectors, whereas *q* is a local position within a vector; the upper rightmost lane has an index number 0, and the lower leftmost lane *W*−1. The advancing direction is determined by comparing the values of the two edge cells, *S*_*V*_[ 0] and *S*_*V*_[*W*−1], such that the difference of the two cells is kept smaller, where the vectorized original *S* matrix is denoted as *S*_*V*_. See our article [25] for further details about the adaptive banded DP algorithm. **b** Data structure for reconstructing the absolute values in the original DP matrices from difference DP matrices, and a schematic view of prefetching a part of the sequences: Each block consists of a set of 32 vectors (or 1024 cells) and is indexed by a block number, *k*. The absolute value of a cell can be calculated as the sum of 64-bit large offset *L*[ *k*], constant 16-bit value (middle delta) *D*[ *q*], and 8-bit small delta *d*[ *p*,*q*] of the cell. Input sequences are converted to a 2-bit encoded string before processing of a block. Subsequences of 32 bases are loaded from the buffers using an unaligned vector load instruction

The adaptive banded DP algorithm [25] is a variant of the traditional banded DP algorithm that reduces the search space by only calculating a part of the DP matrices in which an optimal alignment path is expected to be contained. Instead of determining the band statically, the adaptive banded DP determines the band dynamically as it calculates values in DP matrices (Fig. 2a, b). A forefront vector of constant width departs from the origin, iteratively moves right or down, and forms the band. The forefront vector tries to move away from cells with lower scores (Fig. 2a), ensuring that alignment paths with higher scores are retained in the band at a high probability. The values of the cells in a forefront vector are computed in parallel using SIMD instructions.

So far, we have explained how to fill the cells in the DP matrices, but we have not yet described how to find the position of the cell with the maximum value (score) or how to traceback when base-to-base pairwise alignments are performed.

#### Finding the cell with the maximum value

The absolute values of the cells in DP matrices are basically lost in the difference recurrences. Nonetheless, in semi-global alignment, we need to find the cell with the maximum value so that we can find a position from which the traceback starts; you could say that we need the absolute values for this purpose. Our implementation stores the absolute values of cells in a DP matrix in compressed form.

Before we move on to the data structure that helps us find the maximum value in DP matrices, we will define several terms. First, the band in the DP matrices is divided into smaller “blocks,” each of which contains vectors calculated in 32 successive updates (Fig. 2b). The *k*th block is designated as *B*_*k*_. Assuming that band width *W* is 32, the number of cells in a single block of the DP matrix is 1024. To illustrate how exactly our algorithm stores values, we introduce another coordinate, (*p*,*q*): *p* along the diagonal direction and *q* for the antidiagonal direction in the DP matrix (to specify vectors and lanes; Fig. 2a, b). The location of each vector, which is represented by coordinates (*i*,*j*) of the top right cell of the vector, is being tracked during vector updates. Coordinates (*i*,*j*) and (*p*,*q*) in the different coordinate systems are easily converted into each other. In the text that follows, we use either coordinate system to specify the position of a cell depending on which is more convenient for explanation; *p*-*q* coordinates are mainly used to describe vectors and lanes, whereas *i*-*j* coordinates are used to describe the relation between adjacent cells.

**Fig. 3:**
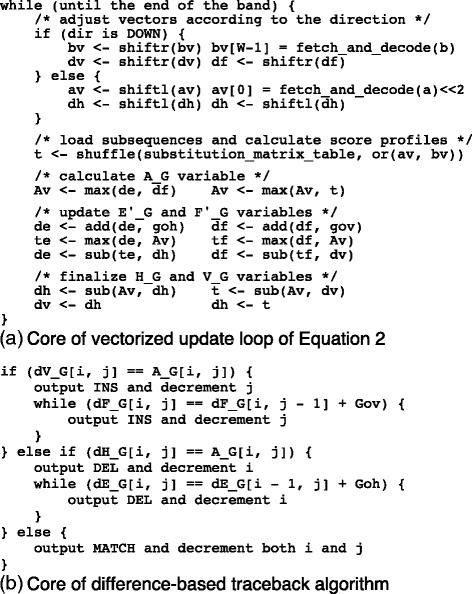
**a** The core of the vectorized update loop: The pseudocode describes a simplified version of the update procedure for the (antidiagonally) vectorized difference recurrence algorithm; dh, dv, df, dh, and Av denote vectors *ΔH*_*G*_, *ΔV*_*G*_, *ΔEG*′, *ΔFG*′, and *A*_*G*_, respectively; av and bv are input sequence vectors that hold 2-bit-encoded nucleotide sequences. The binary operators for vectors—or, max, add, and sub—are bitwise OR and elementwise maximum, addition, and subtraction, respectively. The unary operators, shiftr and shiftl, are elementwise rightward and leftward shift operations, respectively. The shuffle operation (shuffle) takes an element table as the first argument and an index vector as the second argument. **b** The core of the traceback loop: Traceback is executed by comparing difference values. In our implementation, the vertical transition is preferred over both the horizontal and diagonal transition in the DP matrix. The horizontal transition is preferred over the diagonal transition. The diagonal transition is chosen only when the other transitions are impossible (See “Traceback flags and generating alignment path strings” section for details). Note that MATCH indicates the diagonal transition including a mismatch

Figure 3a and b explains how the vectors are updated and how to traceback. Several performance-tuning techniques are omitted in the pseudocode for simplicity. We need to use 32-bit integers or wider to store values in the DP matrices if we store them in a naïve fashion. Given that the difference between the maximum value and minimum value of the cells in a single block fits into 16 bits with the assumption that *M* is small, we subtract (potentially) large offset value *L*[ *k*] from every cell in the block. *L*[ *k*] is a single 64-bit integer stored in memory. After subtraction, the values in the DP matrices can be represented as signed 16-bit integers. We then noticed that the values in the same lane of a block tend to be more similar than values in distant lanes (i.e., *q*-coordinates are distant) because the values of cells tend to be somewhat greater toward the center of the vectors. To further reduce the number of bits required for storing values of cells, we subtracted the *D*[ *q*] constant (as well as *L*[ *k*]) from the cells in the *q*th lane.

In all, we decompose a value of a cell into three values: a 64-bit integer that represents a potentially large offset (*L*[ *k*]), a constant 16-bit signed integer we call a middle delta (*D*[ *q*]), and an 8-bit signed integer that we call a small delta (*d*[ *p*,*q*], where *k*=⌊*p*/32⌋). The absolute value of a cell is the sum of the three values. The small delta values are calculated on every vector update (i.e., on every move of the forefront vector). The maximum values for the small deltas in all the lanes of a block are updated using a SIMD operation. The vector of small deltas represented as a vector of 8-bit integers may overflow with a certain substitution matrix containing large values, but it will not overflow when *M* is relatively small, which is the assumption. We cannot explicitly show how small *M* should be because the algorithm is too complex for rigorous analysis, but we did not see any problems with the combination of parameters typical for the existing long-read aligners.

The initial conditions for the three variables are defined across the *k*=−1 block, which we call the “phantom block,” and its last vector (“phantom vector” at *p*=−1) as shown below. Term |*q*−*W*/2| represents the distance from the center of the band. Coefficient $-(M + G_{e_{H}} + G_{e_{V}})$ in the middle delta denotes the lower bounds of the cell values with an assumption that the center cell has the highest score in each vector. We introduced additional inclined offset |*q*−*W*/2|∗128/*W* into the middle delta to decrease overflow errors, which are often caused by a reduced gradient due to a low-identity region.

$$\begin{array}{*{20}l} {}L[\!-1] &\,=\, 0  \\ D[\!q] &\,=\, -G_{o_{H}} \,+\, |q -\! W/2| * -(M \,+\, G_{e_{H}} \,+\, G_{e_{V}} \,-\, 128\!/\!W)  \\ {}d[\!-1, q] &\,=\, |q - W/2| * -128/W  \end{array} $$

A variant of the X-drop algorithm is implemented using two variables, *d*[ *p*,*q*] and *d*_*max*_[ *p*,*q*]; we compared the difference of the two values (the amount of the drop) with threshold *X*. The large offset is calculated immediately after the last vector of each block becomes available, after which the values in the small-delta vector are shifted accordingly. In the current implementation, the large offset is the average of *W*/4th and 3*W*/4th values in the last vector of a previous block. The large offset, the last small-delta vector, and the maximal small-delta vector are stored for later use in the traceback. All the other small-delta vectors are recalculated on demand in the traceback phase to reduce memory use.

A bonus for combining the difference recurrence relations with the adaptive band algorithm is that this approach eliminates the dependence on the second previous vector (*S*[ *i*−1,*j*−1] in Eq. 1) in the recurrences. The elimination of the dependence on the second previous vector leads to more efficient calculations because it removes a branch in the execution path when the band moves in the same direction twice in a row. Avoiding a branch misprediction penalty, which is quite large (e.g., 16–17 cycles per prediction failure for the Intel Skylake architecture) in the innermost loop has a noticeable impact on computation time.

#### Traceback flags and generating alignment path strings

The final part necessary for finishing the complete pairwise alignment is to generate an alignment path string (a.k.a. an edit path). We introduce another data structure, traceback flags, which are sets of four 32-bit-wide bit vectors that we denote by *M*_*H*_, *M*_*V*_, *M*_*E*_, and *M*_*F*_, where each bit in the vectors represents the possibility of transition (in the traceback) to the corresponding cell. *M*_*H*_[ *i*,*j*] is set iff a horizontal transition is possible from *S*[ *i*,*j*] to *E*[ *i*−1,*j*], and *M*_*E*_[ *i*,*j*] is set iff a horizontal transition is possible from *E*[ *i*,*j*] to *E*[ *i*−1,*j*]. The other two vectors for vertical transitions are defined similarly. The four sets of vectors, *M*_*H*_[ *i*,*j*], *M*_*V*_[ *i*,*j*], *M*_*E*_[ *i*,*j*], and *M*_*F*_[ *i*,*j*], are calculated simultaneously as the DP matrices are being filled. For reasons we will describe shortly, we decided to prefer vertical transitions over horizontal transitions when both transitions are possible. In addition, the diagonal transition is chosen only when both the vertical and horizontal transitions are impossible. Figure 3b describes the traceback algorithm in pseudocode.

We encoded the alignment path in a series of bits. A single bit of “1” represents the vertical transition, “0” the horizontal transition, and “10” the diagonal transition. The final alignment path string is a concatenation of the encoded transitions. You might think that it is impossible to tell “10” from “1” followed by “0”; however, the transition priority we mentioned above enabled us to uniquely determine which interpretation is correct when we read the final alignment path string either from left to right or from right to left. This path string encoding ensures high memory efficiency while retaining several preferable properties: (1) the alignment path string is obtained by concatenating the bit-encoded strings, and (2) the length of any part of the alignment path string of an alignment is the sum of the lengths of the aligned part of one of the aligned sequences and that for the other sequence. That is, the length of the alignment path is calculated from the lengths of the two sequences to be aligned. These properties enable more efficient implementations of several higher-layer algorithms such as breakpoint correction in a split-read alignment. A bonus of this approach is that the traceback implementation can be performed by means of logical operations so that it can reduce the number of branches as compared to a naïve implementation of the SWG algorithm or semi-global alignment algorithm (Eq. 1).

Additionally, we present an efficient method for conversion of the bit-encoded alignment path string to the corresponding CIGAR string. A contiguous deletion is observed as contiguous zeros in the bit-encoded string. Modern processors provide an instruction that counts the contiguous zeros from the least significant (or most significant) bit in a register, which is called “trailing (leading) zero count.” The trailing zero count instruction calculates the length of the deletion block in a single instruction. Determining the length of a region of diagonal transitions only is also possible for the trailing zero count instruction with some trick; taking bitwise XOR with repeated “10” bits, or 0xAAAA...AA, reduces the problem to simple bit counting.

#### Computation of score profile vectors and prefetching sequences

Retrieval of scores from the substitution matrix, or calculation of the score profile vector, can be performed in parallel using the 16-element vector shuffle operation as described by Suzuki and Kasahara [25], but we will give a brief overview. The vector shuffle operation is a parallel table lookup on a SIMD register, where the table of integers of fixed size is “shuffled” with the index vector that has indices between 0 and “vector width minus 1” (15 for the 16-element vector). In order to utilize the shuffle operation to calculate the score profile vector, an index value in the index vector is assumed to be a concatenation of two bases each of which is encoded in 2 bits. The element vector holds a 16-element vector or a flattened substitution matrix. The substitution matrix vector is retained on a SIMD register during the computation, and the index vector is calculated on the fly from 32-base-long subsequences. To mitigate the overhead of the index vector construction, conversion from an ASCII character to a 2-bit encoded integer is done outside the innermost loop, and the conversion is done in parallel using SIMD operations at the beginning of the process for a block. The encoded sequences, both of which are 64 bp, are stored in sequence prefetch buffers (Fig. 2b).

#### Miscellaneous implementation techniques

We describe miscellaneous implementation techniques that exploit common features of modern CPUs. We believe the techniques are applicable to the processors, though, we mainly adopt the x86_64 architectures in the explanation because we only provided implementations for them (SSE4.1 and AVX2 SIMD instructions, available on x86_64 processors by Intel and AMD). Further portability issues that come with the techniques are not discussed here, but discussed in Additional file 1: Section S2.

##### Register usage:

The fill-in and traceback algorithms have small loops that are executed a huge number of times. Therefore, optimization of the register use in the intensive loops is expected to improve the overall calculation performance. Here, we carefully tuned the implementation to make the number of register spill-reload pairs as small as possible and the number of concurrently executable instructions as large as possible (the latter can be accomplished by relaxing a chain of result-to-source register dependences).

The matrix calculation loop of a 32-cell-wide band requires four 32-byte vectors for the four difference variables and a pair of 32-byte vectors for the small delta and maximal small delta variables. These vectors are mapped to a set of twelve 128-bit-wide or six 256-bit-wide SIMD registers. Given that the SSE instruction sets of x86_64 processors give us only sixteen 128-bit-wide registers, we had to implement the fill-in loop carefully to make sure that the number of temporary registers required at the same time does not exceed 4. For this reason, the query sequence buffers were allocated in memory, and the unaligned load operation was adopted to imitate elementwise shift operations on the vectors. The order of several operations from the naïve implementation was reassigned (based on the pseudocode shown in Fig. 3a, b) for the major compiler backends (clang, gcc, and Intel C Compiler) so that we can place scalar and SIMD instructions in an alternating manner. We further optimized the recurrence relations for CPU architectures that do not support three-operand operations. Our implementation for the SSE4.1 instruction sets calculates −*ΔH*_*G*_ instead of *ΔH*_*G*_ using the commutativity of addition, i.e., *A*_*G*_+(−*ΔH*_*G*_) is calculated instead of *A*_*G*_−*ΔH*_*G*_; this approach reduces computation time slightly.

In the traceback loop, we applied similar considerations. Several variables, especially the ones that are referenced few times in the loop (e.g., the length of sequences, indices on the sequences, and the counters for the number of gaps) were moved from the general-purpose registers to SIMD registers. This modification eliminated the register spill/reload pairs almost completely from the innermost loop.

##### Branch predictor consideration:

Modern processors have speculative branch selection and instruction execution mechanisms, called branch prediction. A failure in the branch prediction causes a fairly large execution path recovery penalty (16–17 cycles for Intel Skylake and 18 cycles for AMD Ryzen architectures; [32]). Consequently, we made the control flow of the algorithm as easily predictable as possible, extracting and reassigning simple patterns for each branch for the patternable ones or making the branch probability sufficiently biased for the stochastic ones.

With regards to the fill-in loop, we noticed as a result of observation that the two advancing directions, rightward and downward, repeat alternately in most cases, especially during alignment of high-identity sequence pairs. We unrolled the matrix fill-in loop into 4 blocks, assigning downward direction to the odd ones and rightward to the even ones. This composition eliminates internal branching execution paths for each block and enables efficient streaming instruction execution without control transfer to distant addresses. Rare patterns—two or more contiguous right (or down) advances—are handled by skipping the middle downward-advancing (or rightward-advancing) blocks. Because the longer contiguous identical directions occur more rarely, the 4-fold unrolled loop successfully avoids the pollution of the prediction states due to double or triple contiguous identical directions that sometimes occur between normal zigzag patterns.

The traceback loop was unrolled into three blocks in accordance with the traceback directions—diagonal, vertical, and horizontal—to assign a dedicated prediction state to each inter- and intrablock transition. It was effective at keeping branch prediction states clean, especially for the transitions into the diagonal block (diagonal-to-diagonal, vertical-to-diagonal, and horizontal-to-diagonal) because they have much higher probabilities than the others. The direction-based unrolling was also effective in eliminating unnecessary memory accesses.

## Results

We implemented the proposed affine gap penalty algorithm (Eq. 3) for x86_64 processors using AVX2 SIMD instructions with the optimization techniques described in the “Library implementation” section (libgaba; commit 7648c72). We also provided the difference algorithm implementation without deformation of the recurrences (Eq. 2) or optimization (diff-raw). As comparison baseline, we prepared another implementation, the adaptive banded DP algorithm with affine gap penalty without the use of the difference recurrence relations (non-diff). We chose these algorithms because we wanted to measure the performance gain provided by the difference recurrence relations. The difference recurrences may also be useful for nonbanded DP algorithms, but we excluded such algorithms because they run too slowly when input sequences are long owing to their time complexity of *O*(*n*^2^) in contrast to *O*(*n*) for adaptive banded DP algorithms, where *n* is the length of input sequences. To compare our algorithm with the fastest alignment algorithm for a unit score matrix, we also implemented an alignment algorithm based on Myers’ bit-parallel edit distance algorithm (editdist) with an adaptive band, which is a slightly modified version of the algorithm authored by Kimura [33]. The editdist algorithm is a special case of the adaptive banded DP where the score matrix is a unit matrix. It was expected to run faster than our algorithm because it is a restricted version of our algorithm. The vector width (*W*) was set to 32 in the three affine gap penalty implementations because it is the width of SIMD registers in AVX2 and therefore we can expect the highest efficiency. We set *W* to 64 in the adaptive edit distance algorithm because it is the width of general-purpose registers. Note that the band for editdist is twice as wide as that for the other three algorithms. Bit widths for the DP variables were set to 8 in the two difference algorithms, to 16 in non-diff, and to 1 in editdist. Every algorithm was composed of three stages: matrix fill-in, traceback, and path-to-CIGAR conversion. The benchmark programs were compiled by gcc-5.4.1 with the O3-level optimization with the SIMD instruction enabled (-march=native) and were then executed on an Intel Core i5 6260U processor (Skylake; 2.8 GHz at boost; 4 MB L3 cache) with 32 GB RAM (DDR4; 2,133 MHz) running Ubuntu Linux 16.10.

As input data, query sequence pairs were generated from three runs of Oxford Nanopore MinION reads from the whole-genome sequencing experiments of a human sample, NA12878 (accession No.: FAB45271, FAB42316, and FAB49164; [1]). A thousand subsequence pairs ranging within 25±2 kbp were taken from the SAM file generated by BWA-MEM (version 0.7.15-r1142-dirty) with the ONT 2D setting (-xont2d). The sequence pairs that contained a contiguous gap region longer than 20 bp were filtered out to compare purely the matrix fill-in speed of the algorithms because gaps larger than 20 bp may not be captured by the adaptive banded algorithm at non-negligible probability [25]. Without filtering, algorithms may sometimes terminate alignments long before they reach the ends of input sequences, which would complicate the interpretation of results. The generated input data had the mean read length of 24,691 bp, insertion and deletion rates of 0.014 and 0.091, respectively, and a mismatch rate of 0.061 (Additional file 2). We first determined what percentage of the alignments was largely reproduced by the 32-cell-wide adaptive banded algorithm. The three implementations failed to reproduce the full alignment obtained by BWA-MEM for 2.6% of the pairs in the input. We found that almost all the failed pairs contained homopolymers or tandem-repeat sequences around the point where the alignment was terminated prematurely, suggesting that further improvement is needed to successfully align over 99% of raw reads. The other major failure mode was due to low-identity regions.

Table 1 shows the computation time for aligning the input sequence pairs. The Fill, Trace, Conv, and Total columns present the computation time for filling the DP matrices including time for filling the data for finding the maximum value, computation time for alignment path calculation including the time for finding the position of the cell with the maximum score, the computation time for path-to-CIGAR conversion, and total computation time, respectively. The score parameters employed in the affine gap penalty algorithms were the same as in the BWA-MEM alignment; the match score (*s*(*x*,*y*) when *x*=*y*), the mismatch score (*s*(*x*,*y*) when *x*≠*y*), the gap-open penalties ($G_{o_{H}}$ and $G_{o_{V}}$), and the gap-extension penalties ($G_{e_{H}}$ and $G_{e_{V}}$) were set to 1, −1, 1, and 1, respectively. X-drop threshold *X*=50 was input into the three affine gap penalty algorithms. Maximal edit distance *k* was set to *k*=10,000 for editdist.

**Table 1:** Performance comparison in terms of filling the matrix, traceback, conversion to CIGAR strings, total computation time, and GCUPS

Calc. time (sec.)	Fill	Trace	Conv	Total	GCUPS
Editdist	0.436	0.104	0.076	0.616	7.19
Non-diff	0.565	0.399	0.073	1.037	2.77
Diff-raw	0.516	0.316	0.073	0.905	3.03
Libgaba	0.377	0.097	0.028	0.502	4.15
Edlib	26.0	18.8	0.109	44.9	13.23
SeqAn ED ^a^	77.2				7.97
BWA-MEM global ^b^	354	0.381		355	0.12
BLAST X-drop ^c^	250				0.18
Parasail ^c^	886				0.69

Results of comparison to existing implementations. Four adaptive banded DP implementations of ours (editdist, non-diff, diff-raw, and libgaba; top four rows) and four existing implementations (edlib, SeqAn ED, BWA-MEM global, BLAST X-drop, and Parasail; bottom five rows) were compared. See the main text for the details of the implementations. Columns: The average computation time per cell is shown in the Fill column. The time for traceback and CIGAR string conversion is shown in the Trace and Conv columns. The Total column presents the sum of Fill, Trace, and Conv. The GCUPS column shows the matrix fill-in performance in billion cell updates per second (GCUPS)

^a^The traceback and path-to-CIGAR conversion time for the SeqAn ED were not measured

^b^The path-to-CIGAR conversion field for the BWA-MEM global is blank because the implementation directly generates final CIGAR string

^c^The traceback and the path-to-CIGAR conversion fields are blank because the traceback routines were not implemented for the BLAST X-drop DP and the Farrar’s algorithm implementations that we used

As expected, libgaba runs faster than diff-raw, and diff-raw runs faster than non-diff at any of the Fill, Trace, or Conv steps. The total computation speed for libgaba is 2.1-fold higher than that of non-diff, which has been the fastest implementation for the semi-global alignment of long reads.

Although the libgaba implementation ran slower than editdist in terms of per-cell performance, libgaba ran faster than editdist in terms of per-vector performance. This is remarkable given that editdist can take only the unit score matrix and therefore can be considered a special version of libgaba, and given that editdist would need wider band width to obtain alignments of quality similar to libgaba due to the restriction on the score matrix. To be precise, libgaba spends mere 22 clock cycles per vector update; this speed outperforms editdist. The improvement of the update performance of libgaba can be explained by two factors: a reduction in the critical path length (by ∼ 4 cycles) and the prefetch and conversion of query sequences (∼ 3 cycles). It should also be noted that libgaba makes the maximal use of the available execution units (several scalar units and several SIMD pipelines) in the innermost loop. We observed that libgaba stalls rarely, presumably because all the computation context is kept only on registers; the DP vectors are on the SIMD registers and the others on the general-purpose registers. On the other hand, the editdist implementation generated several register spill/reload pairs that resulted in execution pipeline stalls, which led to a performance loss. The performance of libgaba and editdist on the traceback was significantly faster than that of non-diff and diff-raw. The reason why the performance on the traceback is clustered into two groups may be that the traceback algorithms for both methods are similar in that logical operations are utilized. The speed for the path-to-CIGAR conversion was also improved by a factor of ∼ 2.5 with the proposed bit-counting-based conversion algorithm when compared with the naïve one. We also conducted the same experiment on other machines with different SIMD instruction sets and compilers. The results were generally consistent among all the tested settings, the editdist being the fastest and the libgaba being the second fastest. The machine specifications and complete results are shown in Table S1 and S2, respectively, in Additional file 1: Section S3.

**Fig. 4:**
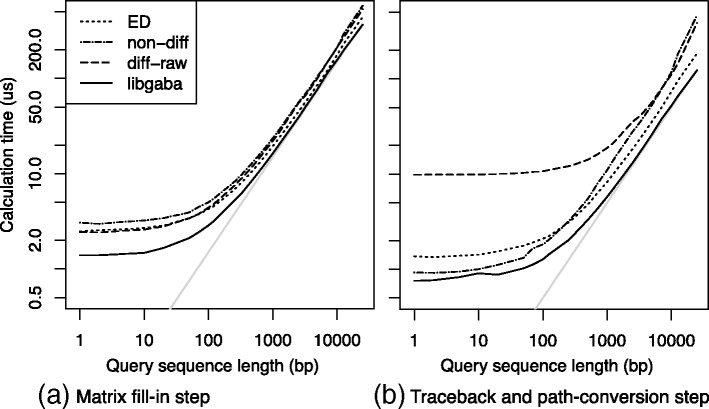
The average computation time in microseconds for (**a**) a single call of the fill function and (**b**) a single call of the trace function and the path-to-CIGAR conversion function, for the four implementations: editdist, non-diff, diff-raw, and libgaba. The length of query sequence pair *l* is tested for each value in the geometric series from 1 to 33,000, with the geometric ratio of approximately 1.25. The gray lines, *t*=0.0145·*l* in the fill and *t*=0.0045·*l* in (**b**), are depicted as regression lines for the results of libgaba

Next, we measured scalability with respect to the length of input sequences. Figure 4a and b shows the average computation time for a single call of the fill or for the traceback and conversion steps for the NA12878 dataset. Sequence length *l* varied between 1 and 25,000 bp except that we cut off the tail longer than 25 kbp in the input sequences. The maximal edit distance was set to *k*=0.6·*l* for editdist. The results were largely consistent with the results in Table 1, as expected. We can see that libgaba scales linearly when sequences are longer than a certain threshold, where the overhead can be ignored as compared to total computation time. The overhead at the fill-in step was presumably due to the extra extension (100–200 bp) until the end of alignment was detected by the X-drop-like heuristic. The large overhead observed at the traceback and conversion step for diff-raw was caused by the additional step involving searching for the cell with the maximum value, which is not a necessary step for other algorithms. We can see that libgaba successfully reduced overhead presumably with the data structure described in the “Library implementation” section despite the large overhead for diff-raw.

### Comparison to existing implementations

We tried to compare our algorithms with previous alignment algorithms used in popular nucleotide sequence aligners, but it was not straightforward as we expected. We found that alignment routines in existing stand-alone long-read aligners or general-purpose alignment libraries cannot be directly compared to our algorithm in a fair manner. Here are some examples: (1) the Farrar’s algorithm [19] is not suited for banded alignment, whereas ours can be used for banded alignment; (2) BWA-MEM [8], NCBI-BLAST+ [34], LAST [9] or NanoBLASTer [35] do not use SIMD, whereas ours use SIMD, although using SIMD (alone) is not our contribution here; (3) GraphMap [12] extensively use the Myers’ edit-distance algorithm that is a kind of data-level parallel algorithm, but the Myers’ algorithm allows us to use the unit score matrix only, although our algorithm accepts a more general score matrix with affine-gap penalty. (4) BLASR [10] relies heavily on seed chaining for reducing the computation time, but does not use SIMD nor bit-parallel algorithms.

Considering those situations, instead of showing a rigid comparison between the existing methods, the baseline algorithm (non-diff), and our algorithm (libgaba), we will show an indirect evidence that the baseline algorithm (non-diff) we used is already well optimized. Other less-optimized SWG algorithms and stand-alone aligners should be easier to optimize, so that they will eventually benefit from our algorithm.

Because our primary contribution is that we doubled (or quadrupled in some cases) the width of SIMD vectorization and that our algorithm still allows tracing back, we decided to evaluate the performance of existing algorithms and ours by measuring the billion cell updates per second (GCUPS), which is how many DP cells are updated per second in the extension alignment.

To that end, we compared the DP matrix calculation performance of our implementation, libgaba and non-diff, the baseline algorithm, with several existing DP matrix calculation routines. We selected five implementations, each of which represents a certain category of existing algorithms: (1) an implementation of the Farrar’s algorithm in the Parasail library (hereafter: Parasail; commit 3d8b4ee; [24]), (2) the global alignment routine in the BWA-MEM algorithm (BWA-MEM global; commit b582816), (3) re-implementation of the BLAST X-drop DP algorithm found in NCBI BLAST+ package (version 2.2.31+), (4, 5) the Myers’ bit-parallel edit distance algorithms [26] in the edlib and the SeqAn libraries (edlib; commit 0c6fe0f; [36] and SeqAn ED; version 1.4.2; [37], respectively). To our knowledge, other tools and libraries for long-read alignment fall largely on one of these categories.

The Parasail implementation we used was a semi-global variant of the full-sized (non-banded) SWG algorithm proposed by Farrar [19]. The implementation uses 256-bit-wide SIMD registers for calculating 16-bit-wide DP cells. The register width and the DP variable width were the same as our non-diff implementation, which achieves the highest parallelism on the machine we used. Although it was unclear whether the implementation also performs well on long nucleotide reads, the Farrar’s algorithm is reported to be the fastest SIMD-vectorized alignment algorithm for protein sequences [19, 23, 24].

The global alignment algorithm in BWA-MEM is a static banded SWG implementation with vertical matrix slicing without SIMD vectorization. Out of the two DP-based alignment implementations (global alignment and semi-global alignment) in BWA-MEM, we chose the global alignment implementation for the following two reasons: (1) The number of the updated cells is easier to calculate for the global alignment implementation; the semi-global alignment routine may stop evaluating cells in the DP-matrix due to the X-drop-like heuristic algorithm (called Z-drop); (2) The semi-global alignment algorithm used in BWA-MEM does not store the whole DP matrix nor the whole traceback information; it only stores the position of the cell where the maximum score is given. This hinders direct comparison with other implementations. The global alignment routine, “ksw_global2” found in ksw.c, calculates the SWG DP matrices with 32-bit-wide variables and stores 8-bit-wide traceback direction flags. We set the bandwidth to the default of the BWA-MEM algorithm, three plus the antidiagonal distance of the two end positions of the alignment for each query-reference pair. This is because the global alignment routine in BWA-MEM is invoked after semi-global alignment finds an approximate region in the DP matrices where an optimal path travels through.

The BLAST X-drop DP is a SWG-based semi-global alignment extension algorithm adopted in the BLAST package. Since the current implementation of the algorithm in the NCBI BLAST+ package was highly complicated in terms of the input and output data structures and the control flows, we used a modified version used in our adaptive banded DP paper [25]. Briefly, we extracted a part of the source code from the NCBI BLAST+ package, optimized the part of the source code for aligning only nucleotide sequences for the benchmark. The control flows were not largely modified form the original implementation, while the score matrix retrieval was simplified by removing branches for protein sequences and position-specific score matrices. The X-drop threshold was set to *X*=70, which is the default value of the blastn program.

The Myers’ edit-distance algorithm utilizes 64-bit general purpose registers to handle 64 DP cells simultaneously. The Myers’ edit-distance algorithm only takes the unit score matrix; it is a special case of the general SWG algorithm. The SeqAn edit-distance implementation calculates a full-sized DP matrix without any heuristic, but again it only takes the unit score matrix. The edlib library adopts a space-reduction technique similar to the BLAST X-drop algorithm for efficiently detecting alignment paths around the diagonal line of the DP matrices. We estimated the number of updated cells by counting the number of calls to “calculateBlock” function, which updates a set of four 64-bit-wide difference vectors of the Myers’ algorithm that consists of a set of information for 64 cells. The number of updated cells were counted using a separate implementation such that the counting does not affect the computation speed measurement. The maximal edit distance parameter was set to *k*=10,000 for the algorithm to capture the full length alignments from the dataset.

We used the same experiment setting as the previous experiment. The 1000 subsequence pairs of the Nanopore dataset of 25 kbp and its corresponding GRCh38 reference subsequence, were input to the five implementations. These implementations were compiled and linked into a single binary. As for the billion cell updates per seconds (GCUPS) metrics, the fastest implementation was the edlib (13.2 GCUPS; Table 1). The SeqAn edit-distance algorithm and our adaptive edit-distance algorithm performed largely equally, at 8.0 and 7.2 GCUPS, respectively. This is what we expected because they accept the unit score matrix only, and therefore they solve only a special case of the SWG problem that the other algorithms solve. Among the SWG implementations (libgaba, BWA-MEM global, and Parasail), the fastest was libgaba (4.15 GCUPS). The Parasail marked 0.69 GCUPS, showing that libgaba fills more cells per second than the state-of-the-art parallel SWG implementation. We speculate that there are two reasons for the performance decline: (1) Cache miss occurs more frequently on long reads due to the large DP matrix, that is, a full-sized DP matrix for 1000 bp sequence pairs becomes 6 MB for the SWG algorithm when 16-bit-wide variable is adopted, which is larger than the last-level cache of current typical processors (e.g., 3 MB for Intel Skylake). (2) The algorithm incorporates dependences between cells inside each SIMD vector, which becomes additional computational overhead compared to the dependence-free vectorization of ours. Both of the BWA-MEM global and the BLAST X-drop DP implementations were much slower than the SIMD-vectorized SWG implementations (0.12 and 0.18 GCUPS, respectively), which is due to the serial (not parallelized) DP matrix calculation. Considering that (1) the implementations write DP cell values (scores) or traceback directions to memory, (2) the implementation accesses memory for loading query and reference bases, the results for the two implementations are likely to represent the performance for a typical serial (non-parallel) SWG implementation.

In all, the edit-distance algorithm in edlib is the fastest in terms of GCUPS. However, the edit-distance algorithm is a special case of the SWG algorithm because it can only take the unit score matrix and because it does not accept affine-gap penalty. Among the SWG algorithm or its variants, libgaba is the fastest in terms of GCUPS. Considering that the theoretical maximum GCUPS after applying 8-way SIMD operations (32-bit-wide variables) to the BLAST X-drop DP alignment algorithm is roughly 0.18×8=1.44, which is far below the GCUPS of libgaba, we conclude that libgaba is the fastest extension alignment algorithm in practice for long reads.

## Discussion

The difference recurrence relations utilize the full width of SIMD instructions available on modern processors using 8-bit integers in most operations during computation of a semi-global alignment. The difference recurrence relations can be easily extended to the global alignment. In addition, we released the implementation of our algorithm as a pure C library so that tool developers can immediately benefit from the difference recurrence relations. Because libgaba is, to the best of our knowledge, the fastest affine gap penalty alignment library suitable for aligning long nucleotide sequences from single-molecule sequencers, we hope that libgaba is incorporated into many existing alignment tools and other tools in the near future.

In addition, our benchmark indicates that the combination of difference recurrence relations and the adaptive banded DP algorithm is highly effective at aligning real reads generated by single-molecule sequencers. Our benchmark also revealed a limitation of the adaptive banded algorithm: large indels (> 20 bp), homopolymers, and tandem repeats must be handled with care in order to calculate more accurate alignments. This may be done by an algorithm in a higher layer. One possible design for the seed-and-extend–based alignment algorithm may be to combine the seed-chaining algorithm with a special extension step. The seed-chaining step enumerates seeds, and then the seeds are chained to estimate the approximate region through which the optimal alignment path should go. The extension step can benefit directly from adaptive banded DP, but a researcher may want to add an extra step that iterates the extension until the alignment covers the full chain when the input sequence contains problematic sequences that cause premature termination of the alignment. Another design may use a nonadaptive (static) banded DP algorithm to overcome problems with tandem-repeat or homopolymer regions by means of static banded DP with a wider band. Be that as it may, we believe that libgaba is already useful for most applications. Indeed, Minimap2 [38] already uses a variant of our difference recurrences, achieving much improved speed over its predecessor. We are also developing a stand-alone aligner.

Alignment of long protein sequences might be also accelerated by the proposed difference recurrence relations. Nevertheless, how to do it efficiently is an open question; our algorithm assumes that the score matrix fits in a single SIMD register [25], but a normal score matrix for amino acids (e.g., 20×20) does not. There might also be an option to combine the difference recurrences with horizontally placed vectors (as in the Farrar’s algorithm), where score profile vectors can be precalculated for each query residue.

Finally, we would like to mention that it is easy to port our algorithm to other flavors of processors such as graphical processing units or manycore processors, where a per-core memory quota or the local memory size is relatively small as compared to the ones in normal CPUs. Our algorithm is also likely to run efficiently on field-programmable gate arrays or application-specific integrated circuits because it can lead to a smaller circuit size of arithmetic units (addition, subtraction, and maximum) and to better timing requirements because of the shorter critical-path length.

## Conclusions

We proposed a novel algorithm “difference recurrence relations” that computes a semi-global Smith-Waterman-Gotoh alignment in a SIMD-friendly manner, as a generalization of existing bit-parallel string comparing algorithms [26, 27]. We also proposed several implementation techniques that are effective on accelerating semi-global alignment algorithms. We released a portable library implementation of our algorithm, libgaba. Our difference recurrence algorithm accelerated constant-width banded DP calculation of nucleotide semi-global alignment. Our library implementation will facilitate accelerating many long-read analysis algorithms that uses pairwise alignment.

## Additional files

Additional file 1 This file contains supplementary sections describing the derivation processes of the difference recurrence relations and the proofs of the bounding formulae (**Section S1**), library design and portability issues (**Section S2**), and benchmark results on different machines (**Section S3**). (PDF 231 kb)

Additional file 2 This archive file contains input nucleotide sequences used in the benchmark and raw outputs of the benchmarking programs. (ZIP 26,419 kb)

## Abbreviations

AVX: Advanced vector eXtension
DP: Dynamic programming
ED: Edit distance
LCS: Longest common substring
GCUPS: Giga cell updates per seconds
SIMD: Single-instruction multiple-data
SSE: Streaming SIMD extension
SWG: Smith-Waterman-Gotoh
