# Supplementary material for: Introducing difference recurrence relations for faster semi-global alignment of long sequences
Source: BMC Bioinformatics. 2018 Feb 19;19(Suppl 1):45. doi: 10.1186/s12859-018-2014-8 (PMC5836832; doi:10.1186/s12859-018-2014-8)
Supplement: Supplementary file 1 — This file contains supplementary sections describing the derivation processes of the difference recurrence relations and the proofs of the bounding formulae (Section S1), library design and portability issues (Section S2), and benchmark results on different machines (Section S3). (PDF 231 kb) [file 12859_2018_2014_MOESM1_ESM.pdf]

S1 Derivation of the difference recurrence relations and proof of the bounding formulae

The semi-global alignment problem (Equation 1 in the main text) is defined for $i \geq 0$ and $j \geq 0$. In the following subsections, we use simplified formulae, where $i \geq 1$ and $j \geq 1$, to describe the process of derivation of the difference recurrence relations and proof of the bounding formulae.

$$S[i,j] = \max \begin{cases} S[i-1,j-1] + s(a_{i-1}, b_{j-1}) \\ E[i-1,j] - G_{e_H} \\ F[i,j-1] - G_{e_V} \end{cases} \tag{4}$$

$$E[i,j] = \max \begin{cases} S[i,j] - G_{o_H} \\ E[i-1,j] - G_{e_H} \end{cases} \tag{5}$$

$$F[i,j] = \max \begin{cases} S[i,j] - G_{o_V} \\ F[i,j-1] - G_{e_V} \end{cases} \tag{6}$$

The initial conditions (where $i = 0$ or $j = 0$) are as follows:

$$S[i,j] = \begin{cases} 0 & (i = 0, j = 0) \\ -G_{o_V} - j \cdot G_{e_V} & (i = 0, j \neq 0) \\ -G_{o_H} - i \cdot G_{e_H} & (i \neq 0, j = 0) \end{cases} \tag{7}$$

$$E[i,j] = \begin{cases} -G_{o_H} - i \cdot G_{e_H} & (j = 0) \\ -\inf & (i = 0) \end{cases} \tag{8}$$

$$F[i,j] = \begin{cases} -G_{o_V} - j \cdot G_{e_V} & (i = 0) \\ -\inf & (j = 0) \end{cases} \tag{9}$$

S1.1 Derivation of the difference recurrence relations

Difference values $\Delta H[i,j]$ are defined for $i \geq 1$, and $\Delta V[i,j]$ for $j \geq 1$. $\Delta E[i,j]$ and $\Delta F[i,j]$ are defined across the cells in the whole DP matrices ($i \geq 0$ and $j \geq 0$).

$$\Delta H[i,j] = S[i,j] - S[i-1,j] \qquad (i \geq 1) \tag{10}$$

$$\Delta V[i,j] = S[i,j] - S[i,j-1] \qquad (j \geq 1) \tag{11}$$

$$\Delta E[i,j] = E[i,j] - S[i,j] \tag{12}$$

$$\Delta F[i,j] = F[i,j] - S[i,j] \tag{13}$$

S1.1.1 ΔH and ΔV

First, we derive the update formulae of ΔH by substituting the right-hand sides of the update formula of S (Eq 4) into $S[i,j]$ in the definition of ΔH (Eq 10).

$$\begin{aligned}
\Delta H[i,j] &= S[i,j] - S[i-1,j] \\
&= \max \left\{ \begin{array}{l} S[i-1,j-1] + s(a_{i-1}, b_{j-1}) \\ E[i-1,j] - G_{e_H} \\ F[i,j-1] - G_{e_V} \end{array} \right\} - S[i-1,j] \\
&= \max \left\{ \begin{array}{l} S[i-1,j-1] - S[i-1,j] + s(a_{i-1}, b_{j-1}) \\ E[i-1,j] - S[i-1,j] - G_{e_H} \\ F[i,j-1] - S[i-1,j] - G_{e_V} \end{array} \right. \\
&= \max \left\{ \begin{array}{l} S[i-1,j-1] - S[i-1,j] + s(a_{i-1}, b_{j-1}) \\ E[i-1,j] - S[i-1,j] - G_{e_H} \\ F[i,j-1] + (S[i,j-1] - S[i,j-1]) + (S[i-1,j-1] - S[i-1,j-1]) - S[i-1,j] - G_{e_V} \end{array} \right. \\
&= \max \left\{ \begin{array}{l} -(S[i-1,j] - S[i-1,j-1]) + s(a_{i-1}, b_{j-1}) \\ (E[i-1,j] - S[i-1,j]) - G_{e_H} \\ (F[i,j-1] - S[i,j-1]) + (S[i,j-1] - S[i-1,j-1]) - (S[i-1,j] - S[i-1,j-1]) - G_{e_V} \end{array} \right. \\
&= \max \left\{ \begin{array}{l} -\Delta V[i-1,j] + s(a_{i-1}, b_{j-1}) \\ \Delta E[i-1,j] - G_{e_H} \\ \Delta F[i,j-1] + \Delta H[i,j-1] - \Delta V[i-1,j] - G_{e_V} \end{array} \right. \\
&= \max \left\{ \begin{array}{l} s(a_{i-1}, b_{j-1}) \\ \Delta E[i-1,j] + \Delta V[i-1,j] - G_{e_H} \\ \Delta F[i,j-1] + \Delta H[i,j-1] - G_{e_V} \end{array} \right\} - \Delta V[i-1,j] \\
&= A[i,j] - \Delta V[i-1,j] \qquad (14)
\end{aligned}$$

A is defined the same as in the main text:

$$A[i,j] = \max \left\{ \begin{array}{l} s(a_{i-1}, b_{j-1}) \\ \Delta E[i-1,j] + \Delta V[i-1,j] - G_{e_H} \\ \Delta F[i,j-1] + \Delta H[i,j-1] - G_{e_V} \end{array} \right. \qquad (15)$$

The update formula for ΔV is derived similarly:

$$\Delta V[i,j] = A[i,j] - \Delta H[i,j-1] \qquad (16)$$

S1.1.2 ΔE and ΔF

$$\begin{aligned}
\Delta E[i,j] &= E[i,j] - S[i,j] \\
&= \max \left\{ \begin{array}{l} S[i,j] - G_{o_H} \\ E[i-1,j] - G_{e_H} \end{array} \right\} - S[i,j] \\
&= \max \left\{ \begin{array}{l} S[i,j] - S[i,j] - G_{o_H} \\ E[i-1,j] - S[i,j] - G_{e_H} \end{array} \right. \\
&= \max \left\{ \begin{array}{l} -G_{o_H} \\ E[i-1,j] + (S[i-1,j] - S[i-1,j]) - S[i,j] - G_{e_H} \end{array} \right. \\
&= \max \left\{ \begin{array}{l} -G_{o_H} \\ (E[i-1,j] - S[i-1,j]) - (S[i,j] - S[i-1,j]) - G_{e_H} \end{array} \right. \\
&= \max \left\{ \begin{array}{l} -G_{o_H} \\ \Delta E[i-1,j] - \Delta H[i,j] - G_{e_H} \end{array} \right. \qquad (17)
\end{aligned}$$

The update formula for ΔF is obtained in a similar fashion:

$$\Delta F[i,j] = \max \left\{ \begin{array}{l} -G_{o_V} \\ \Delta F[i,j-1] - \Delta V[i,j] - G_{e_V} \end{array} \right. \qquad (18)$$

S1.1.3 Initial conditions

These are defined across the cells in the left edge column $i = 0$ and the top edge row $j = 0$. From Equation 4, the initial conditions for ΔH are derived as follows:

$$\Delta H[i,j] = S[i,j] - S[i-1,j] = \left\{ \begin{array}{ll} G_{o_H} + G_{e_H} & (i = 1, j = 0) \\ G_{e_H} & (i \geq 2, j = 0) \end{array} \right. \qquad (19)$$

For the initial conditions for ΔV:

$$\Delta V[i,j] = S[i,j] - S[i,j-1] = \begin{cases} G_{o_V} + G_{e_V} & (i=0, j=1) \\ G_{e_V} & (i=0, j \geq 2) \end{cases} \tag{20}$$

The initial conditions for ΔE and ΔF are defined as shown below. The differences from the $-\inf$ value are clipped to $-G_{o_H}$ and $-G_{o_V}$, which are the minimum values of ΔE and ΔF, as we prove in Section S1.2.3. This modification does not cause a gap penalization error at the edges as pointed out by Flouri *et al.* (2015) because this setting guarantees $E[0,j] - G_{e_H} \leq S[0,j] - G_{o_H} - G_{e_H}$ and $F[i,0] - G_{e_V} \leq S[i,0] - G_{o_V} - G_{e_V}$ for the first updates of ΔE (where $i = 1$) and ΔF ($j = 1$), respectively.

$$\Delta E[i,j] = E[i,j] - S[i,j] = \begin{cases} 0 & (j=0) \\ -G_{o_H} & (i=0) \end{cases} \tag{21}$$

$$\Delta F[i,j] = F[i,j] - S[i,j] = \begin{cases} 0 & (i=0) \\ -G_{o_V} & (j=0) \end{cases} \tag{22}$$

S1.2 Proof of the bounding formulae

We use the following lemma in the proof:

Lemma 1 *If $p = \max\{q, r\}$, then $p \geq q$ and $p \geq r$ always hold.*

S1.2.1 Lower bounds of ΔE and ΔF

According to Equation 5 and Lemma 1, $E[i,j] \geq S[i,j] - G_{o_H}$ always holds for any $i \geq 1$ and $j \geq 1$. Then, the following inequality is instantly derived:

$$\Delta E[i,j] = E[i,j] - S[i,j] \geq -G_{o_H} \tag{23}$$

A similar process is applicable to ΔF:

$$\Delta F[i,j] = F[i,j] - S[i,j] \geq -G_{o_V} \tag{24}$$

S1.2.2 Upper bounds of ΔE and ΔF

From Equation 4 and 5, the following transformation is obtained:

$$\begin{aligned}
\Delta E[i,j] &= E[i,j] - S[i,j] \\
&= \max \begin{cases} S[i,j] - G_{o_H} \\ E[i-1,j] - G_{e_H} \end{cases} - S[i,j] \\
&= \max \begin{cases} -G_{o_H} \\ E[i-1,j] - S[i,j] - G_{e_H} \end{cases} \\
&= \max \begin{cases} -G_{o_H} \\ E[i-1,j] - \max \begin{Bmatrix} S[i-1,j-1] + s(a_{i-1}, b_{j-1}) \\ E[i-1,j] - G_{e_H} \\ F[i,j-1] - G_{e_V} \end{Bmatrix} - G_{e_H} \end{cases} \\
&= \max \begin{cases} -G_{o_H} \\ E[i-1,j] + \min \begin{Bmatrix} -S[i-1,j-1] - s(a_{i-1}, b_{j-1}) \\ -E[i-1,j] + G_{e_H} \\ -F[i,j-1] + G_{e_V} \end{Bmatrix} - G_{e_H} \end{cases} \\
&= \max \begin{cases} -G_{o_H} \\ \min \begin{cases} -S[i-1,j-1] + E[i-1,j] - s(a_{i-1}, b_{j-1}) - G_{e_H} \\ 0 \\ E[i-1,j] - F[i,j-1] - G_{e_H} + G_{e_V} \end{cases} \end{cases} \\
&= \max \begin{cases} -G_{o_H} \\ B_H[i,j] \end{cases}
\end{aligned} \tag{25}$$

A new variable, $B_H[i,j]$, is introduced to replace the ternary minimum block in the formula. The variable is evaluated by means of Lemma 1:

$$B_H[i,j] = \min \begin{Bmatrix} -S[i-1,j-1] + E[i-1,j] - s(a_{i-1}, b_{j-1}) - G_{e_H} \\ 0 \\ E[i-1,j] - F[i,j-1] - G_{e_H} + G_{e_V} \end{Bmatrix} \leq 0 \tag{26}$$

Thus, variable $\Delta E[i,j]$ is bounded from above:

$$\Delta E[i,j] \leq 0 \tag{27}$$

Similar derivation is applicable to ΔF:

$$\Delta F[i,j] \leq 0 \tag{28}$$

S1.2.3 Lower bounds of ΔH and ΔV

From Equations 4 and 5, it follows that $S[i,j] \geq E[i-1,j] - G_{e_H}$ and $E[i,j] \geq S[i,j] - G_{o_H}$ always hold for any i and j, respectively. Thus, the following inequality is obtained according to Lemma 1:

$$S[i,j] \geq E[i-1,j] - G_{e_H} \geq S[i-1,j] - G_{o_H} - G_{e_H} \tag{29}$$

Thus:

$$\Delta H[i,j] = S[i,j] - S[i-1,j] \geq -G_{o_H} - G_{e_H} \tag{30}$$

Similar reasoning applies to ΔV:

$$\Delta V[i,j] = S[i,j] - S[i,j-1] \geq -G_{o_V} - G_{e_V} \tag{31}$$

S1.2.4 Upper bounds of ΔH and ΔV

We first postulate the following lemma:

Lemma 2
$S[i,j] - S[i-k,j] \geq -G_{o_H} - k \cdot G_{e_H}$ for any $i \geq 1$, $j \geq 1$, and $1 \leq k \leq i$. (horizontal)
$S[i,j] - S[i,j-l] \geq -G_{o_V} - l \cdot G_{e_V}$ for any $i \geq 1$, $j \geq 1$, and $1 \leq l \leq j$. (vertical)

Proof: The update formula for S is defined as in Equation 4 for $i \geq 1$ and $j \geq 1$. Then the following transformation logically follows:

$$\begin{aligned}
S[i,j] &= \max \begin{cases} S[i-1,j-1] + s(a_{i-1}, b_{j-1}) \\ E[i-1,j] - G_{e_H} \\ F[i,j-1] - G_{e_V} \end{cases} \\
&= \max \begin{cases} S[i-1,j-1] + s(a_{i-1}, b_{j-1}) \\ \max \left\{ \begin{array}{l} S[i-1,j] - G_{o_H} \\ E[i-2,j] - G_{e_H} \end{array} \right\} - G_{e_H} \\ F[i,j-1] - G_{e_V} \end{cases} \\
&= \max \begin{cases} S[i-1,j-1] + s(a_{i-1}, b_{j-1}) \\ \max \begin{cases} S[i-1,j] - G_{o_H} - G_{e_H} \\ S[i-2,j] - G_{o_H} - 2G_{e_H} \\ \vdots \\ S[1,j] - G_{o_H} - (i-1) \cdot G_{e_H} \\ S[0,j] - G_{o_H} - i \cdot G_{e_H} \end{cases} \\ F[i,j-1] - G_{e_V} \end{cases} \\
&= \max \begin{cases} S[i-1,j-1] + s(a_{i-1}, b_{j-1}) \\ \max_{1 \leq k \leq i} S[i-k,j] - G_{o_H} - k \cdot G_{e_H} \\ F[i,j-1] - G_{e_V} \end{cases} \\
&\geq \max_{1 \leq k \leq i} S[i-k,j] - G_{o_H} - k \cdot G_{e_H}
\end{aligned} \tag{32}$$

In the last transformation, we used Lemma 1. Hence,

$$S[i,j] - S[i-k,j] \geq -G_{o_H} - k \cdot G_{e_H} \quad \text{where } 1 \leq k \leq i \tag{33}$$

Similarly, for the vertical:

$$S[i,j] - S[i,j-l] \geq -G_{o_V} - l \cdot G_{e_V} \quad \text{where } 1 \leq l \leq j \tag{34}$$

Using Lemma 2, we next prove the following lemma, which postulates that the diagonal difference is always bounded by the maximum value in the substitution matrix:

Lemma 3 *$S[i,j] - S[i-1,j-1] \le M$ for any $i \ge 1$ and $j \ge 1$*

Proof: Let us assume that the following inequality holds for any $1 \le s \le i$ and $1 \le t \le j$ except for the $s = i$ and $t = j$ pair:

$$S[s,t] - S[s-1,t-1] \le M \tag{35}$$

Then, we evaluate the upper bound of the $S[i,j]$ value using the following transformation and three resulting terms (i)–(iii):

$$\begin{aligned}
S[i,j] &= \max \begin{cases} S[i-1,j-1] + s(a_{i-1}, b_{j-1}) \\ E[i-1,j] - G_{e_H} \\ F[i,j-1] - G_{e_V} \end{cases} \\
&= \max \begin{cases} S[i-1,j-1] + s(a_{i-1}, b_{j-1}) \\ \max \begin{cases} S[i-1,j] - G_{o_H} - G_{e_H} \\ S[i-2,j] - G_{o_H} - 2G_{e_H} \\ \vdots \\ S[1,j] - G_{o_H} - (i-1) \cdot G_{e_H} \\ S[0,j] - G_{o_H} - i \cdot G_{e_H} \end{cases} \\ \max \begin{cases} S[i,j-1] - G_{o_V} - G_{e_V} \\ S[i,j-2] - G_{o_V} - 2G_{e_V} \\ \vdots \\ S[i,1] - G_{o_V} - (j-1) \cdot G_{e_V} \\ S[i,0] - G_{o_V} - j \cdot G_{e_V} \end{cases} \end{cases} \\
&= \max \begin{cases} S[i-1,j-1] + s(a_{i-1}, b_{j-1}) & \dots \text{(i)} \\ \max_{1 \le k \le i} S[i-k,j] - G_{o_H} - k \cdot G_{e_H} & \dots \text{(ii)} \\ \max_{1 \le l \le j} S[i,j-l] - G_{o_V} - l \cdot G_{e_V} & \dots \text{(iii)} \end{cases}
\end{aligned} \tag{36}$$

Case (i): When the first term (i) reaches the maximum of the ternary maximum block, the equation below holds for $S[i,j]$ and $S[i-1,j-1]$:

$$S[i,j] - S[i-1,j-1] \le \max_{p,q} s(p,q) = M \tag{37}$$

Thus, Equation 35 is also true at (i,j) in this case.

Case (ii): When the second term (ii) reaches the maximum, the case is further subdivided into the following two subcases (a) and (b):

$$\begin{aligned}
S[i,j] &= \max_{1 \le k \le i} S[i-k,j] - G_{o_H} - k \cdot G_{e_H} \\
&= \max \begin{cases} S[0,j] - G_{o_H} - i \cdot G_{e_H} & (k = i) & \text{(a)} \\ \max_{1 \le k \le i-1} S[i-k,j] - G_{o_H} - k \cdot G_{e_H} & (1 \le k \le i-1) & \text{(b)} \end{cases}
\end{aligned} \tag{38}$$

The first term (a), where $k = i$, is evaluated as follows by means of Equation 35, Lemma 2(horizontal), and the maximum of the initial conditions for ΔV (Eq 20):

$$\begin{aligned}
S[0,j] - G_{o_H} - i \cdot G_{e_H} &= S[0,j] + (S[0,j-1] - S[0,j-1]) + (S[i-1,j-1] - S[i-1,j-1]) - G_{o_H} - i \cdot G_{e_H} \\
&= (S[0,j] - S[0,j-1]) + (S[0,j-1] - S[i-1,j-1]) + S[i-1,j-1] - G_{o_H} - i \cdot G_{e_H} \\
&\le -G_{e_V} + G_{o_H} + (i-1) \cdot G_{e_H} + S[i-1,j-1] - G_{o_H} - i \cdot G_{e_H} \\
&= S[i-1,j-1] - G_{e_H} - G_{e_V}
\end{aligned} \tag{39}$$

The second term (b), where $1 \le k \le i-1$, is evaluated as follows:

$$\begin{aligned}
\max_{1 \le k \le i-1} S[i-k,j] - G_{o_H} - k \cdot G_{e_H} &= \max_{1 \le k \le i-1} \begin{array}{l} S[i-k,j] + (S[i-k-1,j-1] - S[i-k-1,j-1]) \\ +(S[i-1,j-1] - S[i-1,j-1]) - G_{o_H} - k \cdot G_{e_H} \end{array} \\
&= \max_{1 \le k \le i-1} \begin{array}{l} (S[i-k,j] - S[i-k-1,j-1]) + (S[i-k-1,j-1] - S[i-1,j-1]) \\ +S[i-1,j-1] - G_{o_H} - k \cdot G_{e_H} \end{array} \\
&\le \max_{1 \le k \le i-1} M + G_{o_H} + k \cdot G_{e_H} + S[i-1,j-1] - G_{o_H} - k \cdot G_{e_H} \\
&= \max_{1 \le k \le i-1} M + S[i-1,j-1]
\end{aligned} \tag{40}$$

Thus, Equation 35 also holds at (i, j) in this case:

$$\begin{aligned}
S[i,j] - S[i-1,j-1] &\leq \max\left\{\begin{array}{l} S[i-1,j-1] - G_{e_H} - G_{e_V} \\ \max_{1\leq k\leq i} M + S[i-1,j-1] \end{array}\right\} - S[i-1,j-1] \\
&= \max\left\{\begin{array}{l} -G_{e_H} - G_{e_V} \\ \max_{1\leq k\leq i} M \end{array}\right. \\
&= M
\end{aligned} \tag{41}$$

Case (iii): Similar to Case (ii), with Lemma 2 (vertical).

S1.2.5

Next, we evaluate another antidiagonal interlayer difference (i.e., the difference between a pair of cells at the same coordinates in the DP matrices) to state another lemma:

Lemma 4
$E[i-1,j] - S[i,j-1] \leq M + 2G_{e_H}$ for any $i \geq 1,\ j \geq 1$.
$F[i,j-1] - S[i-1,j] \leq M + 2G_{e_V}$ for any $i \geq 1,\ j \geq 1$.

Proof:

$$\begin{aligned}
E[i-1,j] &= \max\left\{\begin{array}{l} S[i-1,j] - G_{o_H} \\ E[i-2,j] - G_{e_H} \end{array}\right. \\
&= \max\left\{\begin{array}{l} S[i-1,j] - G_{o_H} \\ S[i-2,j] - G_{o_H} - G_{e_H} \\ \vdots \\ S[1,j] - G_{o_H} - (i-2)\cdot G_{e_H} \\ S[0,j] - G_{o_H} - (i-1)\cdot G_{e_H} \end{array}\right. \\
&= \max_{0\leq k\leq i-1} S[i-k-1,j] - G_{o_H} - k\cdot G_{e_H} \\
&= \max\left\{\begin{array}{lll} S[0,j] - G_{o_H} - (i-1)\cdot G_{e_H} & (k = i-1) & \text{(i)} \\ \max_{0\leq k\leq i-2} S[i-k-1,j] - G_{o_H} - k\cdot G_{e_H} & (0 \leq k \leq i-2) & \text{(ii)} \end{array}\right.
\end{aligned} \tag{42}$$

Case (i): The first term (i) is evaluated from above by means of the maximum value of the initial conditions for ΔV and Lemma 2 as shown below:

$$\begin{aligned}
S[0,j] - G_{o_H} - (i-1)\cdot G_{e_H} &= S[0,j] + (S[0,j-1] - S[0,j-1]) + (S[i,j-1] - S[i,j-1]) - G_{o_H} - (i-1)\cdot G_{e_H} \\
&= (S[0,j] - S[0,j-1]) + (S[0,j-1] - S[i,j-1]) + S[i,j-1] - G_{o_H} - (i-1)\cdot G_{e_H} \\
&\leq -G_{e_V} + G_{o_H} + i\cdot G_{e_H} + S[i,j-1] - G_{o_H} - (i-1)\cdot G_{e_H} \\
&= -G_{e_V} + G_{e_H} + S[i,j-1]
\end{aligned} \tag{43}$$

Case (ii): The second term (ii) is evaluated using Lemma 2 and 3:

$$\begin{aligned}
\max_{0\leq k\leq i-2} S[i-k-1,j] - G_{o_H} - k\cdot G_{e_H} &= \max_{0\leq k\leq i-2} \begin{array}{l} S[i-k-1,j] + (S[i-k-2,j-1] - S[i-k-2,j-1]) \\ +(S[i,j-1] - S[i,j-1]) - G_{o_H} - k\cdot G_{e_H} \end{array} \\
&= \max_{0\leq k\leq i-2} \begin{array}{l} (S[i-k-1,j] - S[i-k-2,j-1]) + (S[i-k-2,j-1] - S[i,j-1])) \\ +S[i,j-1] - G_{o_H} - k\cdot G_{e_H} \end{array} \\
&\leq \max_{0\leq k\leq i-2} M + G_{o_H} + (k+2)\cdot G_{e_H} + S[i,j-1] - G_{o_H} - k\cdot G_{e_H} \\
&= M + 2G_{e_H} + S[i,j-1]
\end{aligned} \tag{44}$$

Hence,

$$\begin{aligned}
E[i-1,j] - S[i,j-1] &\leq \max\left\{\begin{array}{l} -G_{e_V} + G_{e_H} + S[i,j-1] \\ M + 2G_{e_H} + S[i,j-1] \end{array}\right\} - S[i,j-1] \\
&= \max\left\{\begin{array}{l} -G_{e_V} + G_{e_H} \\ M + 2G_{e_H} \end{array}\right. \\
&= M + 2G_{e_H}
\end{aligned} \tag{45}$$

The proof is similar for F:

$$F[i,j-1] - S[i-1,j] \leq M + 2G_{e_V} \tag{46}$$

S1.2.6

Finally, we derive the upper bound of ΔH using Equation 27 and Lemmas 2 and 4:

$$\begin{aligned}
\Delta H[i,j] &= S[i,j] - S[i-1,j] \\
&= \max \left\{ \begin{array}{l} S[i-1,j-1] + s(a_{i-1}, b_{j-1}) \\ E[i-1,j] - G_{e_H} \\ F[i,j-1] - G_{e_V} \end{array} \right\} - S[i-1,j] \\
&= \max \left\{ \begin{array}{l} -(S[i-1,j] - S[i-1,j-1]) + s(a_{i-1}, b_{j-1}) \\ (E[i-1,j] - S[i-1,j]) - G_{e_H} \\ (F[i,j-1] - S[i-1,j]) - G_{e_V} \end{array} \right. \\
&\leq \max \left\{ \begin{array}{l} M + G_{o_V} + G_{e_V} \\ -G_{e_H} \\ M + G_{e_V} \end{array} \right. \\
&= M + G_{o_V} + G_{e_V}
\end{aligned} \tag{47}$$

Hence,

$$\Delta H[i,j] \leq M + G_{o_V} + G_{e_V} \tag{48}$$

Similarly, for $\Delta V[i,j]$:

$$\Delta V[i,j] \leq M + G_{o_H} + G_{e_H} \tag{49}$$

S1.3 Derivation of the offsetted difference recurrence relation

The definitions of the difference DP matrices with an offset and of the substitution matrix with an offset are the same as in the main text:

$$\Delta H_G[i,j] = \Delta H[i,j] + G_{o_H} + G_{e_H} \tag{50}$$
$$\Delta V_G[i,j] = \Delta V[i,j] + G_{o_V} + G_{e_V} \tag{51}$$
$$\Delta E'_G[i,j] = \Delta E[i,j] + \Delta V[i,j] + G_{o_H} + G_{o_V} + G_{e_V} \tag{52}$$
$$\Delta F'_G[i,j] = \Delta F[i,j] + \Delta H[i,j] + G_{o_V} + G_{o_H} + G_{e_H} \tag{53}$$
$$s_G(x,y) = s(x,y) + G_{o_H} + G_{e_H} + G_{o_V} + G_{e_V} \tag{54}$$

S1.3.1 ΔH_G and ΔV_G

$$\begin{aligned}
\Delta H_G[i,j] &= \Delta H[i,j] + G_{o_H} + G_{e_H} \\
&= A[i,j] - \Delta V[i-1,j] + G_{o_H} + G_{e_H} \\
&= \max\left\{\begin{array}{l} s(a_{i-1}, b_{j-1}) \\ \Delta E[i-1,j] + \Delta V[i-1,j] - G_{e_H} \\ \Delta F[i,j-1] + \Delta H[i,j-1] - G_{e_V} \end{array}\right\} - \Delta V[i-1,j] + G_{o_H} + G_{e_H} \\
&= \max\left\{\begin{array}{l} s(a_{i-1}, b_{j-1}) \\ \Delta E[i-1,j] + \Delta V[i-1,j] - G_{e_H} \\ \Delta F[i,j-1] + \Delta H[i,j-1] - G_{e_V} \end{array}\right\} - \Delta V[i-1,j] + G_{o_H} + G_{e_H} \\
&\quad + ((G_{o_H} + G_{e_H} + G_{o_V} + G_{e_V}) - (G_{o_H} + G_{e_H} + G_{o_V} + G_{e_V})) \\
&= \max\left\{\begin{array}{l} s(a_{i-1}, b_{j-1}) + G_{o_H} + G_{e_H} + G_{o_V} + G_{e_V} \\ \Delta E[i-1,j] + \Delta V[i-1,j] + G_{o_H} + G_{o_V} + G_{e_V} \\ \Delta F[i,j-1] + \Delta H[i,j-1] + G_{o_H} + G_{e_H} + G_{o_V} \end{array}\right\} - \Delta V[i-1,j] - G_{o_V} - G_{e_V} \\
&= \max\left\{\begin{array}{l} s_G(a_{i-1}, b_{j-1}) \\ \Delta E'_G[i,j] \\ \Delta F'_G[i,j] \end{array}\right\} - \Delta V_G[i-1,j] \\
&= A_G[i,j] - \Delta V_G[i-1,j]
\end{aligned} \tag{55}$$

$A_G[i,j]$ is defined as in the main text:

$$A_G[i,j] = A[i,j] + G_{o_H} + G_{e_H} + G_{o_V} + G_{e_V} = \max\left\{\begin{array}{l} s_G(a_{i-1}, b_{j-1}) \\ \Delta E'_G[i-1,j] \\ \Delta F'_G[i,j-1] \end{array}\right. \tag{56}$$

Similarly, for $\Delta V_G[i,j]$:

$$\Delta V_G[i,j] = A_G[i,j] - \Delta H_G[i,j-1] \tag{57}$$

S1.3.2 $\Delta E'_G$ and $\Delta F'_G$

$$\begin{aligned}
\Delta E'_G[i,j] &= \Delta E[i,j] + \Delta V[i,j] + G_{o_H} + G_{o_V} + G_{e_V} \\
&= \max\left\{\begin{array}{l} -G_{o_H} \\ \Delta E[i-1,j] - \Delta H[i,j] - G_{e_H} \end{array}\right\} + \Delta V[i,j] + G_{o_H} + G_{o_V} + G_{e_V} \\
&= \max\left\{\begin{array}{l} -G_{o_H} \\ \Delta E[i-1,j] - A[i,j] + \Delta V[i-1,j] - G_{e_H} \end{array}\right\} + A[i,j] - \Delta H[i,j-1] + G_{o_H} + G_{o_V} + G_{e_V} \\
&= \max\left\{\begin{array}{l} A[i,j] + G_{o_V} + G_{e_V} \\ \Delta E[i-1,j] + \Delta V[i-1,j] + G_{o_H} - G_{e_H} + G_{o_V} + G_{e_V} \end{array}\right\} - \Delta H[i,j-1] \\
&= \max\left\{\begin{array}{l} A[i,j] + G_{o_H} + G_{e_H} + G_{o_V} + G_{e_V} \\ \Delta E[i-1,j] + \Delta V[i-1,j] + 2G_{o_H} + G_{o_V} + G_{e_V} \end{array}\right\} - \Delta H[i,j-1] - G_{o_H} - G_{e_H} \\
&= \max\left\{\begin{array}{l} A_G[i,j] \\ \Delta E'_G[i-1,j] + G_{o_H} \end{array}\right\} - \Delta H_G[i,j-1]
\end{aligned} \tag{58}$$

Likewise, for $\Delta F'_G[i,j]$:

$$\Delta F'_G[i,j] = \max\left\{\begin{array}{l} A_G[i,j] \\ \Delta F'_G[i,j-1] + G_{o_V} \end{array}\right\} - \Delta V_G[i-1,j] \tag{59}$$

S1.4 Bounding formulae for difference DP matrices with an offset

S1.4.1 ΔH_G and ΔV_G

The bounds of ΔH_G instantly follow from Inequality 30 and 48 after addition of the gap penalty offsets:

$$0 \leq \Delta H_G[i,j] \leq M + G_{o_H} + G_{e_H} + G_{o_V} + G_{e_V} \tag{60}$$

Similarly, for ΔV_G, from Inequality 31 and 49, we get

$$0 \leq \Delta V_G[i,j] \leq M + G_{o_H} + G_{e_H} + G_{o_V} + G_{e_V} \tag{61}$$

S1.4.2 $\Delta E'_G$ and $\Delta F'_G$

From Lemmas 2 and 4, the upper bound of $\Delta E'_G$ is derived in the following way:

$$\begin{aligned}
\Delta E'_G[i,j] &= \Delta E[i,j] + \Delta V[i,j] + G_{o_H} + G_{o_V} + G_{e_V} \\
&= E[i,j] - S[i,j-1] + G_{o_H} + G_{o_V} + G_{e_V} \\
&= \max \left\{ \begin{array}{l} S[i,j] - G_{o_H} \\ E[i-1,j] - G_{e_H} \end{array} \right\} - S[i,j-1] + G_{o_H} + G_{o_V} + G_{e_V} \\
&= \max \left\{ \begin{array}{l} S[i,j] - S[i,j-1] + G_{o_V} + G_{e_V} \\ E[i-1,j] - S[i,j-1] + G_{o_H} - G_{e_H} + G_{o_V} + G_{e_V} \end{array} \right. \\
&\leq \max \left\{ \begin{array}{l} M + G_{o_H} + G_{e_H} + G_{o_V} + G_{e_V} \\ M + G_{o_H} + G_{e_H} + G_{o_V} + G_{e_V} \end{array} \right. \\
&= M + G_{o_H} + G_{e_H} + G_{o_V} + G_{e_V}
\end{aligned} \tag{62}$$

The lower bound is instantly obtained from Inequality 23 via addition of the gap penalty offsets. The complete bounding formulae are shown below:

$$0 \leq \Delta E'_G[i,j] \leq M + G_{o_H} + G_{e_H} + G_{o_V} + G_{e_V} \tag{63}$$

In a similar fashion for $\Delta F'_G$:

$$0 \leq \Delta F'_G[i,j] \leq M + G_{o_H} + G_{e_H} + G_{o_V} + G_{e_V} \tag{64}$$

S2 Library design and implementation details

The library is implemented in the pure C programming language in an object-oriented manner. Given that the C language does not explicitly support classes or object-specialized functions (class methods), we designed APIs to take a pointer to an object instance as the first argument to treat a C function call as a class method call. The return object and other reference arguments are also handled by pointers, and thus we consider an object instance and the pointer to an instance equivalent in the context of argument passing in the description below.

S2.1 Target architectures

The library implies 64-bit little-endian architectures with 128- or 256-bit-wide SIMD instruction and unaligned load/store capability. The possible targets are x86_64 with SSE4.1 or AVX2 (Intel Corporation (2016), Advanced Micro Devices Inc. (2013)), AArch64 with NEON (ARM Ltd. (2017)), and 64-bit PowerPC with AltiVec instructions (in little-endian mode; OpenPOWER Foundation (2017)). The library currently supports only the x86_64 architecture, whereas the architecture-dependent operations are separated from the algorithm implementation into headers, which are specified to provide several abstract vector types (e.g., v32i8_t, v16i8_t, and v2i64_t), operations on them, and several bit manipulation operations like popcnt and trailing zero count. We provided two variants, SSE4.1 and AVX2, in the current implementation.

S2.2 API design

The library is supposed to be a component of seed-and-extend–style alignment algorithms, that is, the library supports only the semi-global extension alignment (not local or global alignment). We also regard the library as thread-safe, with a global immutable configuration context and the thread-local DP matrix context. The global context is initialized with a set of substitution matrix, gap penalties, and several other parameters like the X-drop threshold. The local context is generated from the global object, inheriting the configuration and initializing its own DP matrix and memory arena. The memory management in the local context is not designed to be interthread-portable; thus, the users must not pass any derived object of a local context (an object that is returned from a function that takes a local context as the first argument) to another local context. The following code snippet shows the signature of the two context initialization functions: global and local.[1] The two boundary arguments, alim and blim, are added to inform the library at the tail address of the user space, which is utilized to index reverse-complemented sequences. A sequence pointer that leads to an address larger than the boundary is

[1] Because this document is not a manual for the library, the detailed description of the parameters and behavior of the APIs is omitted.

treated as a "phantom sequence," and the reverse-complemented one at the mirrored address is used. This behavior enables library users to save memory for sequences, where only forward ones are kept in memory and reverse-complemented ones are distinguished by mirrored pointers.

```
/* global context initialization function */
gaba_t *gaba_init(
    gaba_params_t const *params);

/* local context initialization function */
gaba_dp_t *gaba_dp_init(
    gaba_t const *global_context,
    uint8_t const *alim,
    uint8_t const *blim);
```

The alignment function takes three arguments—"tail object" of the previous band and reference side and query side sequences—and tries to extend alignment after the tail object with two input sequences. The function returns a new tail object with at least one of the following three states: X-drop termination, reference-side sequence depletion, or query side sequence depletion. This behavior enables us to handle the input sequence as a linear concatenation (or list) of subsequences. This feature is introduced to make the API compatible with circular or graphically structured genomic sequences like string graphs. The fill_root function is provided to start the banded alignment, which internally creates an "empty" tail object and pass it to the normal matrix fill-in function. The following code snippet is a simple linear-to-linear alignment calculation with a 32-base-long "margin" sequence, which is generally an array of zeros, to ensure that the ends of the input sequences are covered by the band, where r and q are pointers to the reference side and the query side sequence segments, and m is a pointer to the margin, whereas rsp and qsp are respectively start positions in the reference and query. Note that each subsequence is distinguished by a "sequence ID," which is a 32-bit unsigned number uniquely assigned to the subsequences.

```
/* build section structs */
gaba_section_t rsec = gaba_build_section(0, r, 0x800000000000);
gaba_section_t qsec = gaba_build_section(0, q, 0x800000000000);
gaba_section_t msec = gaba_build_section(0, m, 0x800000000000);

/* keep current pointers on rp and qp */
gaba_section_t const *rp = &rsec, *qp = &qsec;

/* fill-in the body of the banded matrix */
gaba_fill_t *f = gaba_dp_fill_root(
    dp, rp, rsp, qp, qsp);

/* keep section with the maximum cell value on m */
gaba_fill_t *m = f;

/* fill-in the tail of the banded matrix */
uint32_t flag = GABA_STATUS_TERM;
do {
    if (f->status & GABA_STATUS_UPDATE_A) {
        rp = &msec;
    }
    if (f->status & GABA_STATUS_UPDATE_B) {
        qp = &msec;
    }
    flag |= f->status & (
        GABA_STATUS_UPDATE_A | GABA_STATUS_UPDATE_B
    );
    f = gaba_dp_fill(dp, f, rp, qp);
    m = (f->max > m->max)? f : m;
} while (!(flag & f->status));

/* fill-in stage is done, m holds block with the maximum */
```

The tail object also keeps information on the maximum-scoring cell in the band. The search_max function returns a set of reference side and query side sequence IDs and local positions within the subsequences.

```
gaba_pos_pair_t gaba_dp_search_max(
    gaba_dp_t *local_context,
    gaba_fill_t const *section);
```

The traceback function is designed to handle seed-and-extend–style alignment efficiently accepting two tail objects: forward and reverse. The resulting paths are concatenated at their root in opposite directions to generate the complete (full-length) alignment path. It is also possible to insert short matches between the two roots as the seed sequence of the alignment. Passing tail objects to the function results in an alignment object, which contains the score of the alignment, alignment path, and a list of the corresponding sections and breakpoint coordinates for them.

```
gaba_alignment_t *gaba_dp_trace(
    gaba_dp_t *local_context,
    gaba_fill_t const *fw_tail,
    gaba_fill_t const *rv_tail,
    gaba_trace_params_t const *params);
```

S3 Results for SSE 4.1 variants

Our libgaba and the other SIMD implementations (non-diff and diff-raw) support both SSE4.1 128-bit-wide and AVX2 256-bit-wide vectorizations. We compared the performance of the two vectorization variants on several combinations of CPU microarchitectures and compilers. The detailed configurations of the four machines are listed in Table S1, and the results are shown in Table S2. We tested two additional variants in instruction encoding, REX- and VEX-prefix encoded, for the SSE4.1 ones to investigate the effect of the instruction encoding on performance.

The results showed that the libgaba implementation was generally as fast as or slightly slower than editdist in all the other tested environments when the AVX2 instruction was enabled, regardless of the compiler and its version, indicating that the design of the algorithm and data structures and tuning applied to the library were generally effective for x86_64 processors. It is also noteworthy that the acceleration ratio trends were roughly consistent with the results on the Skylake system, which are presented in the main text.

Table S1: Specifications of the four systems.

CPU arch.	Model	Clock	DRAM speed	OS	Compilers	Description
Ivy Bridge	Core i5-3230M	2.6GHz	DDR3-1600	Mac OS X 10.11.6	Apple clang, clang-4.0, gcc-5.4.0	MacBook Pro Retina 13" early 2013
Haswell	Xeon E5-2670 v3	2.3GHz	DDR3-1600	Red Hat Enterprise Linux 6.8	gcc-4.9.3, Intel C compiler 16.0.3 (icc-16.0.3)	Shirokane3 at Human Genome Center
Skylake	Core i5-6260U	2.8GHz @ boost	DDR4-2133	Ubuntu 16.04.2 LTS	clang-3.8, gcc-5.4.1	Intel NUC 6i5SYH
Zen	Ryzen 7-1700	3.7GHz @ boost	DDR4-2400	Ubuntu 17.04	clang-3.8, gcc-6.3.0	Personal Desktop

The systems are distinguished by their CPU microarchitectures and the other components (DRAM, OS, and available compilers). The Haswell system is a single node of the Shirokane3 cluster at the Human Genome Center, the University of Tokyo. All the others are personal.

References

Advanced Micro Devices Inc. (2013). AMD64 architecture programmer's manual volume 1-5. http://developer.amd.com/resources/developer-guides-manuals/.

ARM Ltd. (2017). ARMv8-A reference manual (issue B.a). http://infocenter.arm.com/help/index.jsp?topic=/com.arm.doc.ddi0487b.a/index.html.

Flouri, T., Kobert, K., Rognes, T., and Stamatakis, A. (2015). Are all global alignment algorithms and implementations correct? *bioRxiv*. doi: 10.1101/031500.

Intel Corporation (2016). Intel® 64 and IA-32 architectures software developer manuals. https://software.intel.com/en-us/articles/intel-sdm/.

OpenPOWER Foundation (2017). Power instruction set architecture (ISA) version 3.0B. https://openpowerfoundation.org/?resource_lib=power-isa-version-3-0/.

Table S2: Results of the speed benchmark of REX- and VEX-encoded SSE4.1 and of AVX2 variants on four systems.

Implementation	CPU arch.	Model	Clock	DRAM speed	Vectorization	Compiler	Arch. flags	Fill (ms)	Trace (ms)	Conv(ms)	Total (ms)
editdist	Ivy Bridge	Core i5-3230M	2.6GHz	DDR3-1600	64-bit GP reg.	Apple clang	-march=native	0.462	0.135	0.089	0.686
					64-bit GP reg.	clang-4.0	-march=native	0.460	0.134	0.089	0.683
					64-bit GP reg.	gcc-5.4.0	-march=native	0.463	0.136	0.090	0.689
	Haswell	Xeon E5-2670 v3	2.3GHz	DDR3-1600	64-bit GP reg.	gcc-4.9.3	-march=native	0.373	0.103	0.107	0.583
					64-bit GP reg.	icc-16.0.3	-march=native	0.372	0.103	0.108	0.583
	Skylake	Core i5-6260U	2.8GHz	DDR4-2133	64-bit GP reg.	clang-3.8	-march=native	0.456	0.110	0.080	0.646
					64-bit GP reg.	gcc-5.4.1	-march=native	0.438	0.106	0.077	0.621
	Zen	Ryzen 7-1700	3.7GHz	DDR4-2400	64-bit GP reg.	clang-3.8	-march=native	0.279	0.082	0.095	0.455
					64-bit GP reg.	gcc-6.3.0	-march=native	0.278	0.082	0.094	0.454
non-diff	Ivy Bridge	Core i5-3230M	2.6GHz	DDR3-1600	SSE4.1	Apple clang	-msse4.2	1.266	0.702	0.081	2.049
					SSE4.1 (VEX)	Apple clang	-march=native	1.189	0.701	0.075	1.965
					SSE4.1	clang-4.0	-msse4.2	1.280	0.705	0.082	2.067
					SSE4.1 (VEX)	clang-4.0	-march=native	1.179	0.701	0.074	1.954
					SSE4.1	gcc-5.4.0	-msse4.2	1.313	0.718	0.083	2.115
					SSE4.1 (VEX)	gcc-5.4.0	-march=native	1.196	0.704	0.076	1.976
	Haswell	Xeon E5-2670 v3	2.3GHz	DDR3-1600	SSE4.1	gcc-4.9.3	-msse4.2	0.979	0.238	0.103	1.320
					SSE4.1 (VEX)	gcc-4.9.3	-mavx	0.978	0.245	0.104	1.326
					AVX2	gcc-4.9.3	-march=native	0.593	0.239	0.101	0.933
					SSE4.1	icc-16.0.3	-msse4.2	1.006	0.250	0.106	1.362
					SSE4.1 (VEX)	icc-16.0.3	-mavx	0.983	0.239	0.105	1.327
					AVX2	icc-16.0.3	-march=native	0.593	0.244	0.100	0.937
	Skylake	Core i5-6260U	2.8GHz	DDR4-2133	SSE4.1	clang-3.8	-msse4.2	1.208	0.391	0.074	1.673
					SSE4.1 (VEX)	clang-3.8	-mavx	0.913	0.389	0.072	1.374
					AVX2	clang-3.8	-march=native	0.558	0.388	0.074	1.020
					SSE4.1	gcc-5.4.1	-msse4.2	1.184	0.404	0.073	1.661
					SSE4.1 (VEX)	gcc-5.4.1	-mavx	1.054	0.404	0.076	1.533
					AVX2	gcc-5.4.1	-march=native	0.577	0.410	0.075	1.061
	Zen	Ryzen 7-1700	3.7GHz	DDR4-2400	SSE4.1	clang-3.8	-msse4.2	0.765	0.363	0.095	1.223
					SSE4.1 (VEX)	clang-3.8	-mavx	0.719	0.362	0.094	1.175
					AVX2	clang-3.8	-march=native	0.589	0.368	0.093	1.050
					SSE4.1	gcc-6.3.0	-msse4.2	0.722	0.353	0.095	1.170
					SSE4.1 (VEX)	gcc-6.3.0	-mavx	0.720	0.362	0.094	1.176
					AVX2	gcc-6.3.0	-march=native	0.590	0.356	0.093	1.039
diff-raw	Ivy Bridge	Core i5-3230M	2.6GHz	DDR3-1600	SSE4.1	Apple clang	-msse4.2	0.786	0.500	0.078	1.364
					SSE4.1 (VEX)	Apple clang	-march=native	0.746	0.500	0.068	1.314
					SSE4.1	clang-4.0	-msse4.2	0.787	0.500	0.078	1.365
					SSE4.1 (VEX)	clang-4.0	-march=native	0.764	0.503	0.070	1.337
					SSE4.1	gcc-5.4.0	-msse4.2	0.888	0.547	0.095	1.530
					SSE4.1 (VEX)	gcc-5.4.0	-march=native	0.762	0.505	0.070	1.338
	Haswell	Xeon E5-2670 v3	2.3GHz	DDR-1600	SSE4.1	gcc-4.9.3	-msse4.2	0.654	0.207	0.107	0.968
					SSE4.1 (VEX)	gcc-4.9.3	-mavx	0.614	0.214	0.103	0.931
					AVX2	gcc-4.9.3	-march=native	0.504	0.206	0.102	0.813
					SSE4.1	icc-16.0.3	-msse4.2	0.668	0.217	0.109	0.994
					SSE4.1 (VEX)	icc-16.0.3	-mavx	0.607	0.207	0.104	0.918
					AVX2	icc-16.0.3	-march=native	0.511	0.212	0.102	0.825
	Skylake	Core i5-6260U	2.8GHz	DDR4-2133	SSE4.1	clang-3.8	-msse4.2	0.885	0.325	0.077	1.287
					SSE4.1 (VEX)	clang-3.8	-mavx	0.664	0.316	0.073	1.053
					AVX2	clang-3.8	-march=native	0.525	0.328	0.076	0.929
					SSE4.1	gcc-5.4.1	-msse4.2	0.860	0.327	0.075	1.262
					SSE4.1 (VEX)	gcc-5.4.1	-mavx	0.708	0.329	0.076	1.113
					AVX2	gcc-5.4.1	-march=native	0.535	0.327	0.077	0.938
	Zen	Ryzen 7-1700	3.7GHz	DDR4-2400	SSE4.1	clang-3.8	-msse4.2	0.497	0.135	0.093	0.725
					SSE4.1 (VEX)	clang-3.8	-mavx	0.463	0.131	0.096	0.690
					AVX2	clang-3.8	-march=native	0.461	0.143	0.095	0.699
					SSE4.1	gcc-6.3.0	-msse4.2	0.495	0.143	0.093	0.730
					SSE4.1 (VEX)	gcc-6.3.0	-mavx	0.465	0.137	0.096	0.698
					AVX2	gcc-6.3.0	-march=native	0.461	0.144	0.095	0.701
libgaba	Ivy Bridge	Core i5-3230M	2.6GHz	DDR3-1600	SSE4.1	Apple clang	-msse4.2	0.531	0.091	0.032	0.654
					SSE4.1 (VEX)	Apple clang	-march=native	0.528	0.090	0.033	0.651
					SSE4.1	clang-4.0	-msse4.2	0.530	0.091	0.032	0.653
					SSE4.1 (VEX)	clang-4.0	-march=native	0.518	0.088	0.033	0.638
					SSE4.1	gcc-5.4.0	-msse4.2	0.610	0.099	0.034	0.743
					SSE4.1 (VEX)	gcc-5.4.0	-march=native	0.523	0.089	0.033	0.646
	Haswell	Xeon E5-2670 v3	2.3GHz	DDR3-1600	SSE4.1	gcc-4.9.3	-msse4.2	0.611	0.088	0.031	0.730
					SSE4.1 (VEX)	gcc-4.9.3	-mavx	0.533	0.091	0.032	0.655
					AVX2	gcc-4.9.3	-march=native	0.386	0.094	0.029	0.509
					SSE4.1	icc-16.0.3	-msse4.2	0.622	0.089	0.032	0.743
					SSE4.1 (VEX)	icc-16.0.3	-mavx	0.543	0.090	0.032	0.665
					AVX2	icc-16.0.3	-march=native	0.384	0.094	0.029	0.506
	Skylake	Core i5-6260U	2.8GHz	DDR4-2133	SSE4.1	clang-3.8	-msse4.2	0.723	0.099	0.033	0.856
					SSE4.1 (VEX)	clang-3.8	-mavx	0.508	0.099	0.034	0.640
					AVX2	clang-3.8	-march=native	0.395	0.102	0.030	0.527
					SSE4.1	gcc-5.4.1	-msse4.2	0.720	0.098	0.033	0.851
					SSE4.1 (VEX)	gcc-5.4.1	-mavx	0.512	0.100	0.033	0.645
					AVX2	gcc-5.4.1	-march=native	0.377	0.098	0.028	0.503
	Zen	Ryzen 7-1700	3.7GHz	DDR4-2400	SSE4.1	clang-3.8	-msse4.2	0.440	0.069	0.022	0.532
					SSE4.1 (VEX)	clang-3.8	-mavx	0.404	0.072	0.022	0.498
					AVX2	clang-3.8	-march=native	0.374	0.075	0.022	0.471
					SSE4.1	gcc-6.3.0	-msse4.2	0.439	0.069	0.022	0.531
					SSE4.1 (VEX)	gcc-6.3.0	-mavx	0.403	0.072	0.022	0.496
					AVX2	gcc-6.3.0	-march=native	0.374	0.075	0.022	0.471

The benchmark setting and the definitions of the Fill, Trace, Conv, and Total columns are the same as in the main text. The detailed specifications of the systems are listed in Table S1. The boldfaced numbers show the fastest variant for each pair system–stage (column). The REX-encoded binaries were generated using architecture flag -msse4.2 to enable both SSE4.1 instructions and the popcnt instruction (included in SSE 4.2). The VEX-encoded binaries were generated with the -mavx flag, where SSE 4.1 instructions were used in the explicit vectorizations and several AVX instructions (e.g., vmovaps) were employed in the automatic loop vectorization and some libc functions like memset and memcpy. The -march=native flag was applied to enable all the available instructions and optimizations as the fastest baselines on each system.
