# Supplementary figures and images for: Introducing difference recurrence relations for faster semi-global alignment of long sequences
Source: BMC Bioinformatics. 2018 Feb 19;19(Suppl 1):45. doi: 10.1186/s12859-018-2014-8 (PMC5836832; doi:10.1186/s12859-018-2014-8)

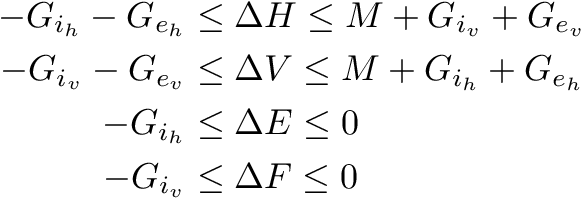

Supplement: Supplementary file 2 — This archive file contains input nucleotide sequences used in the benchmark and raw outputs of the benchmarking programs. (ZIP 26,419 kb) [file 12859_2018_2014_MOESM2_ESM.zip › supplementary_data/benchmark_codes/fig/diffbound.png]

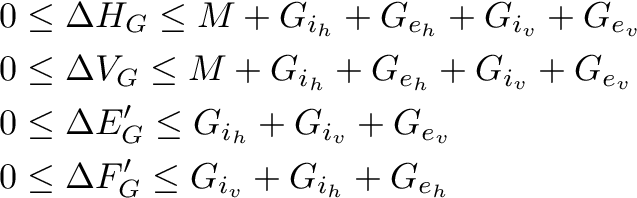

Supplement: Supplementary file 2 — This archive file contains input nucleotide sequences used in the benchmark and raw outputs of the benchmarking programs. (ZIP 26,419 kb) [file 12859_2018_2014_MOESM2_ESM.zip › supplementary_data/benchmark_codes/fig/diffbound2.png]

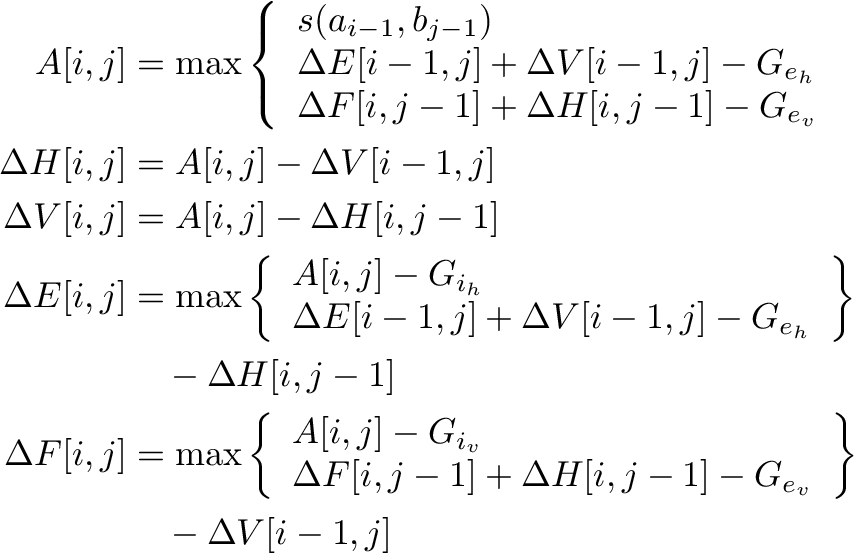

Supplement: Supplementary file 2 — This archive file contains input nucleotide sequences used in the benchmark and raw outputs of the benchmarking programs. (ZIP 26,419 kb) [file 12859_2018_2014_MOESM2_ESM.zip › supplementary_data/benchmark_codes/fig/diffrec.png]

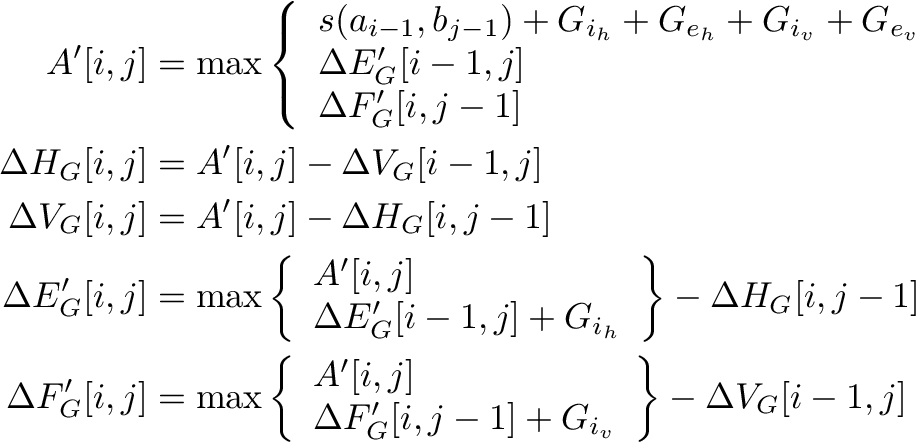

Supplement: Supplementary file 2 — This archive file contains input nucleotide sequences used in the benchmark and raw outputs of the benchmarking programs. (ZIP 26,419 kb) [file 12859_2018_2014_MOESM2_ESM.zip › supplementary_data/benchmark_codes/fig/diffrec2.png]

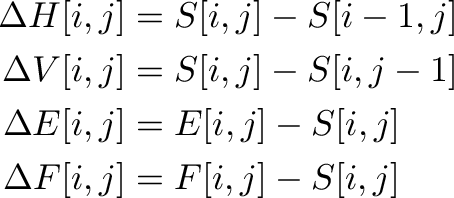

Supplement: Supplementary file 2 — This archive file contains input nucleotide sequences used in the benchmark and raw outputs of the benchmarking programs. (ZIP 26,419 kb) [file 12859_2018_2014_MOESM2_ESM.zip › supplementary_data/benchmark_codes/fig/diffvals.png]

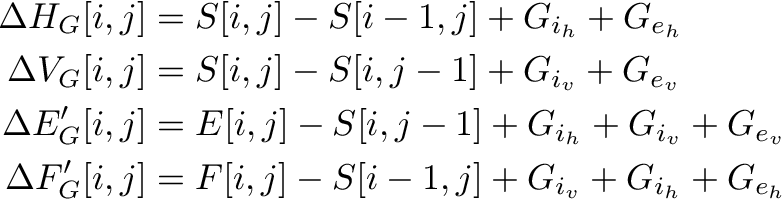

Supplement: Supplementary file 2 — This archive file contains input nucleotide sequences used in the benchmark and raw outputs of the benchmarking programs. (ZIP 26,419 kb) [file 12859_2018_2014_MOESM2_ESM.zip › supplementary_data/benchmark_codes/fig/diffvals2.png]

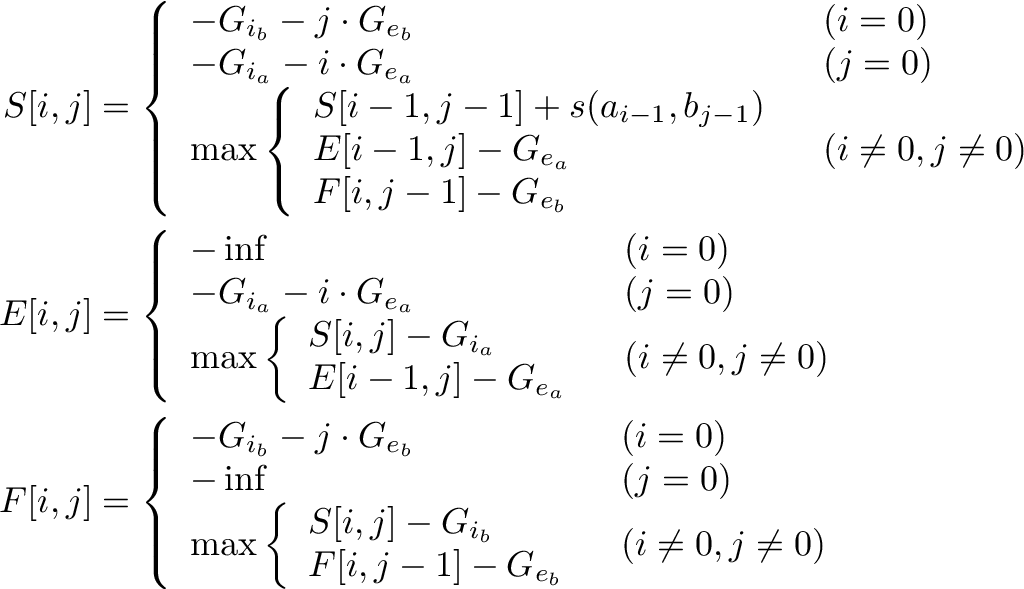

Supplement: Supplementary file 2 — This archive file contains input nucleotide sequences used in the benchmark and raw outputs of the benchmarking programs. (ZIP 26,419 kb) [file 12859_2018_2014_MOESM2_ESM.zip › supplementary_data/benchmark_codes/fig/swg.png]
